# Fennoscandian freshwater control on Greenland hydroclimate shifts at the onset of the Younger Dryas

**DOI:** 10.1038/ncomms9939

**Published:** 2015-11-17

**Authors:** Francesco Muschitiello, Francesco S. R. Pausata, Jenny E. Watson, Rienk H. Smittenberg, Abubakr A. M. Salih, Stephen J. Brooks, Nicola J. Whitehouse, Artemis Karlatou-Charalampopoulou, Barbara Wohlfarth

**Affiliations:** 1 Department of Geological Sciences and Bolin Centre for Climate Research, Stockholm University, SE-10691 Stockholm, Sweden; 2 Department of Meteorology and Bolin Centre for Climate Research, Stockholm University, SE-10691 Stockholm, Sweden; 3 School of Geography, Archaeology and Palaeoecology, Queen's University Belfast, Belfast BT7 1NN, UK; 4 Department of Entomology, Natural History Museum, Cromwell Road, London SW7 5BD, UK; 5 School of Geography, Earth and Environmental Sciences, Plymouth University, Drake Circus, Plymouth PL4 8AA, UK; 6 Department of Geology—Quaternary Sciences, Lund University, Sölvegatan, 12,22362 Lund, Sweden

## Abstract

Sources and timing of freshwater forcing relative to hydroclimate shifts recorded in Greenland ice cores at the onset of Younger Dryas, ∼12,800 years ago, remain speculative. Here we show that progressive Fennoscandian Ice Sheet (FIS) melting 13,100–12,880 years ago generates a hydroclimate dipole with drier–colder conditions in Northern Europe and wetter–warmer conditions in Greenland. FIS melting culminates 12,880 years ago synchronously with the start of Greenland Stadial 1 and a large-scale hydroclimate transition lasting ∼180 years. Transient climate model simulations forced with FIS freshwater reproduce the initial hydroclimate dipole through sea-ice feedbacks in the Nordic Seas. The transition is attributed to the export of excess sea ice to the subpolar North Atlantic and a subsequent southward shift of the westerly winds. We suggest that North Atlantic hydroclimate sensitivity to FIS freshwater can explain the pace and sign of shifts recorded in Greenland at the climate transition into the Younger Dryas.

## Abstract (web summary)

Resolving the sources and timing of freshwater forcing during the Last Termination is key to evaluate the North Atlantic Ocean response to freshwater perturbations. Here, the authors that the North Atlantic climate system was highly sensitive to Scandinavian meltwater discharge shortly before the Younger Dryas.

## Graphical abstract

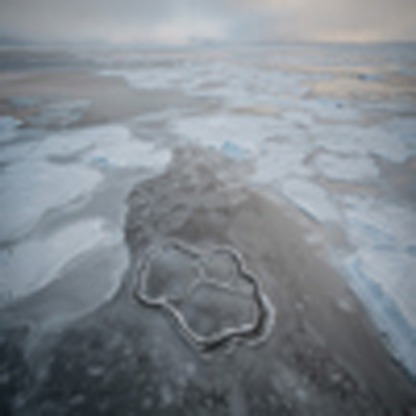

The Younger Dryas cold stadial (YD; **∼**12,800–11,650 year BP) is the latest major large-scale climate shift in the North Atlantic domain, providing an exceptional natural laboratory to improve our understanding of rapid climate change. The conventional explanation for the YD involves a catastrophic meltwater outburst from the Laurentian Ice Sheet into the North Atlantic that triggered a widespread reorganization of the atmosphere–ocean system^1^,^2^,^3^. However, the chronological thread linking Laurentian freshwater events and the timing of abrupt hydroclimate shifts observed in template records such as Greenland ice cores still remains equivocal^3^,^4^,^5^,^6^,^7^.

Recent studies^8^,^9^,^10^ have moved away from the classical flood hypothesis and demonstrated that gradual freshwater input from the Fennoscandian Ice Sheet (FIS)—rather than from the Laurentian Ice Sheet—may have been sufficient to trigger cold stadials during the last glacial cycle. Through southward storm track shifts, mediated by build-up of sea ice in the Nordic Seas, FIS meltwater fluxes at the end of interstadials emerge as a critical factor for reconciling the timing and amplitude of the rapid interstadial/stadial transitions observed in Greenland. Nevertheless, the coherency of these processes during the last glacial–interglacial transition, the Last Termination, is largely unexplored. Furthermore, it remains an open question as to what extent the North Atlantic hydroclimate patterns responded to FIS freshwater forcing at the inception of the YD.

Here we combine a new hydroclimate reconstruction from Northern Europe with transient climate model simulations to show that North Atlantic atmospheric circulation was sensitive to FIS meltwater at the end of the Last Termination. Our conclusions are critical for the interpretation of Greenland ice-core records and could help to target future paleoclimate model simulations.

## Results

### FIS meltwater signal propagation into hydroclimate records

**Figure 1:**
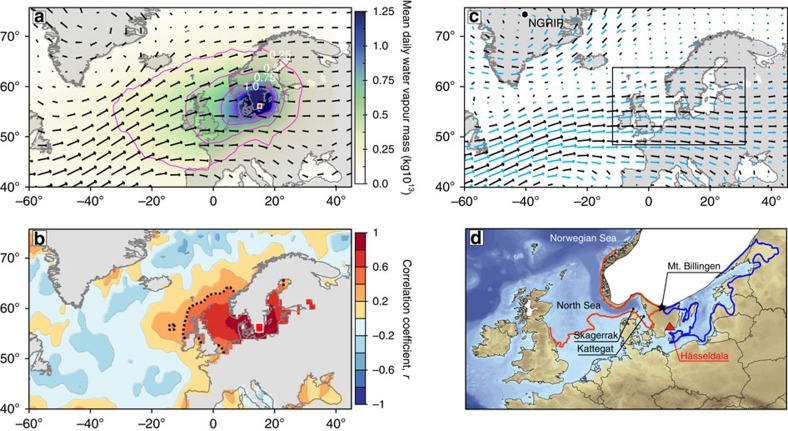
Modern and past summer atmospheric circulation over Southern Sweden and the North Atlantic. (**a**) Precipitation source distribution showing averaged daily water vapour mass (1998–2008) transported to the location of Hässeldala, target of precipitation (red box). Results are plotted together with climatology of 850 hPa wind fields in the North Atlantic region (NCEP/NCAR; 1979–2009). (**b**) Climatological field correlations between specific humidity (HadCRUH; 1974–2003) at Hässeldala (red box) and SSTs (HadSST1), showing the relation between moisture mass transported to the target and surface water temperatures at the marine moisture sources. Significance levels are indicated by black dashed lines (95%). (**c**) Climatological 850 hPa wind fields during the regional Allerød (black arrows) and Younger Dryas (cyan arrows) pollen zones as modelled in the TraCE simulations^33^,^44^. Location of the NGRIP ice core is indicated. The black box highlights the area shown in d. (**d**) Location of Hässeldala (red triangle) in Southern Sweden and paleogeographic setting. The extension of the Baltic Ice Lake (blue dashed line) and palaeogeography of the North Sea (orange dashed line) during the Allerød pollen zone are also displayed. The black star indicates the location of the Baltic Ice Lake's outlet at Mt. Billingen in south-central Sweden^14^.

In this study, we reconstruct the regional sequence of hydroclimate events at the onset of the YD using the hydrogen isotope composition of lipid biomarker records from Hässeldala Port (HÄ) lake sediments, Southern Sweden. HÄ is a small ancient lake^11^,^12^ located along the south coast of Sweden (56°16′ N; 15°03′ E) and downwind of the primary drainage route of the FIS (Fig. 1). Under modern conditions, precipitation is delivered to HÄ by the prevailing westerly winds mainly from the North Sea, the Skagerrak–Kattegat basin and from local continental sources (Fig. 1a). On the other hand, moist air from the Baltic Sea only occurs under exceptionally warm surface water conditions^13^. At multidecadal scales, the amount of moisture transported to HÄ from marine sources primarily depends on surface-water temperatures, which control water-to-air vapour fluxes (Fig. 1b). During the Last Termination, moisture transport from the ice-dammed Baltic Ice Lake^14^ was probably negligible owing to low surface temperatures of glacial lake waters inhibiting moisture fluxes, and to the dominant westerly winds^15^ (Fig. 1c). Therefore, hydroclimate proxies from HÄ sediments are ideally suited for reconstructing the signal of FIS meltwater flux to the adjacent seaboard of the Nordic Seas (Fig. 1d) integrated as isotopic depletions in the hydrogen stable isotope composition of the target precipitation.

### Proxy records and meltwater reconstruction

We analysed the δ*D* composition of *n*-C_21_ and *n*-C_27-29-31_ alkanes (Methods; Supplementary Figs 1 and 2; Supplementary Data 1), which in HÄ sediments are representative components of distinct aquatic and terrestrial sources, respectively (Supplementary Discussion). δ*D* values of aquatic (δ*D*_aq_) and terrestrial (δ*D*_terr_) *n*-alkanes are established indicators of the isotopic composition of summer precipitation δ*D* (refs 16, 17), which is controlled—at mid-to-high latitudes—by condensation temperature and moisture source composition^18^. Furthermore, δ*D*_terr_ can offset δ*D*_aq_ owing to evaporative enrichment (Δδ*D*_terr−aq_) due to the combined effect of soil evaporation and leaf water transpiration, evapotranspiration, thus serving as an indicator of moisture availability and relative humidity^19^ (Supplementary Figs 3 and 4).

The interpretation of the biomarker record is supported by quantitative summer temperature estimations based on fossil chironomids (Methods; Supplementary Data 1). Moreover, HÄ's chronology is based on an age-depth model of 49 AMS ^14^C dates covering ∼4,000 years (Methods; Supplementary Figs 5–7; Supplementary Table 1). After the recent synchronization of the ^14^C and ice-core time scales using the common cosmogenic radionuclide variations^20^ (Supplementary Methods), the age-depth model allows for a consistent and accurate comparison to Greenland stratigraphic events. Hence, HÄ proxies are here compared to NGRIP δ^18^O records^21^ and all ice-core ages are hereafter reported as calibrated ^14^C years before 1950 (BP).

To better decipher the regional hydroclimate expressions in Greenland and Northern Europe and their potential links, we also compare the NGRIP deuterium excess *d* (ref. 22) and GRIP snow accumulation rates^23^ with HÄ δ*D*_aq_ and Δδ*D*_terr−aq_ records. The *d*-excess provides information on the Greenland's moisture source and summer precipitation rates^24^. The δ*D*_aq_ is interpreted here as a proxy for changes in precipitation-source δ*D* after accounting for local hydrologic and vegetation effects and following correction for global ice volume and isotope fractionation factors (δ*D*_corr_; Supplementary Methods; Supplementary Fig. 8). The δ*D*_corr_ records shifts in distillation of water vapour associated with the marine moisture source^25^—primarily the North Sea and the Skagerrak–Kattegat at the temporal resolution of our records—and associated with regional land surface recycling of evaporated moisture^26^. The δ*D*_corr_ is largely controlled by FIS loss through the introduction of isotopically depleted meltwater in the moisture-source area. Moreover, meltwater discharge causes decreases in source seawater salinity, surface temperatures, in source moisture uptake and rainout during transport^18^, all resulting in more negative δ*D* of precipitation and drier air reaching HÄ. Hence, we regard the δ*D*_corr_ and Δδ*D*_terr-aq_ records as qualitative indicators of FIS freshwater supply to the adjacent Nordic Seas.

### Data interpretation

**Figure 2:**
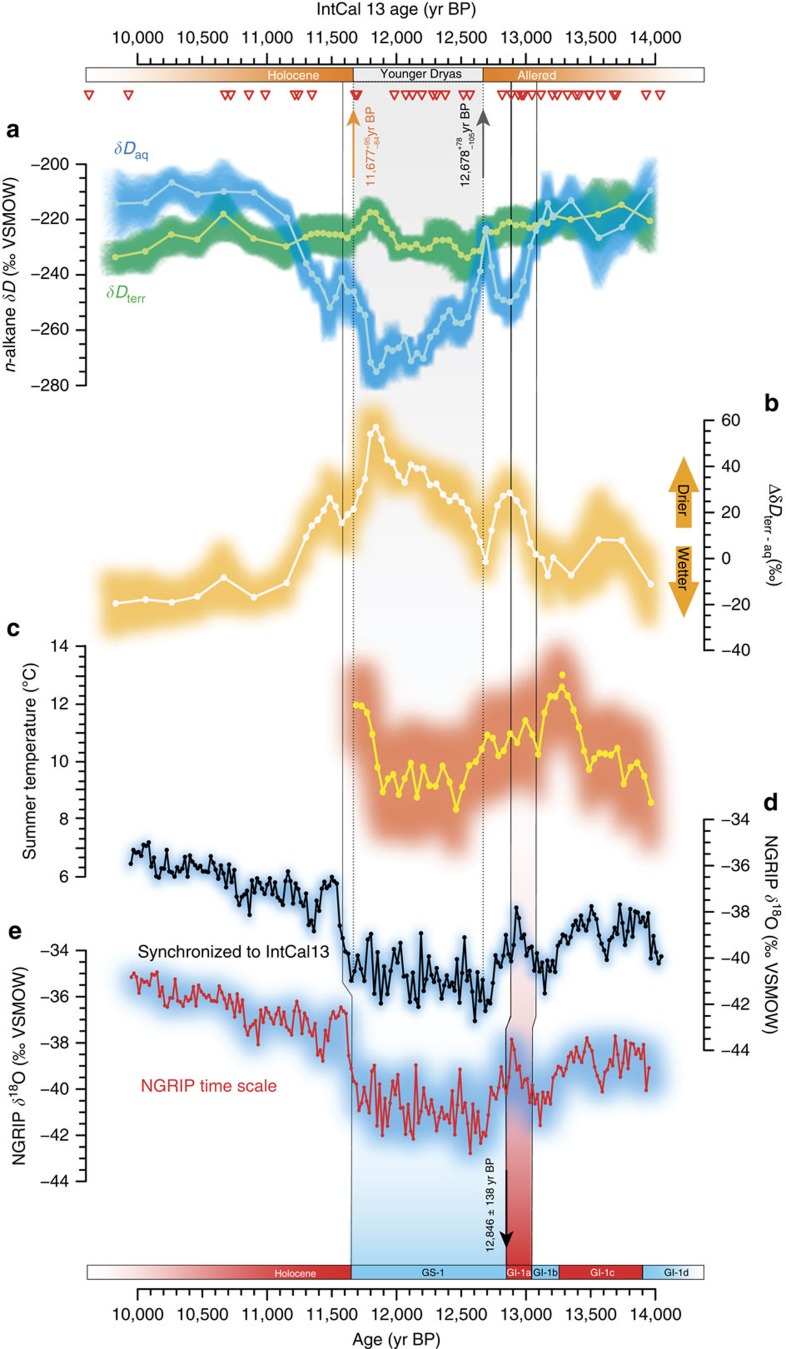
Paleoclimate proxy data from Hässeldala and the Greenland ice core record. δ*D* values of (**a**) *n*-C_21_ (aquatic plants δ*D*_aq_; blue) and weighted average of δ*D* values of *n*-C_27–29–31_ based on relative *n*-alkane abundances (higher terrestrial plants δ*D*_terr_; green), as well as (**b**) terrestrial evapotranspiration (Δδ*D*_terr-aq_) and (**c**) chironomid-based summer temperatures during the regional YD pollen zone at Hässeldala compared to (**d**,**e**) the NGRIP δ^18^O record^21^. The NGRIP record is plotted both on its original time scale and on the IntCal13 time scale (see text for details) after synchronization between the ice-core ^10^Be and tree-ring ^14^C time scales^20^. Beyond 13,500 years BP, which is the limit of the synchronization between the time scales, we reset the GICC05 cumulative counting error^21^. Red triangles denote ^14^C chronological constraints used in the final age-depth model. All records are presented with shadings indicating empirical 95% uncertainty bounds based on analytical and age-model errors. The Hässeldala pollen stratigraphy and Greenland climate events based on the GICC05 (after converting b2k age to BP) are indicated at the top and in the bottom, respectively. The timing of the major excursion in δ*D*_aq_ values at 13090±37 year BP was estimated using a Bayesian change point procedure^45^.

The HÄ δ*D*_aq_ and Δδ*D*_terr−aq_ records show a remarkable two-step decrease and increase, respectively, starting shortly before the onset of the YD as defined in the pollenstratigraphy^11^ (Fig. 2). Similarly, after a ∼300-year long summer warming of up to 4 °C during the Allerød pollen zone (AL), chironomid–inferred temperatures indicate a prominent two-step cooling preceding the start of the YD (Fig. 2). In the first step δ*D*_aq_ values start to decrease by 25‰ at 13,090±37 year BP (±1*σ*) and reach an isotopic minimum at 12,883±35 year BP. This δ*D*_aq_ decline coincides with a 27‰ rise in Δδ*D*_terr-aq_, peaking at 12,883±35 year BP, and a ∼2 °C decrease in summer temperatures (Fig. 2), suggesting substantially drier and colder summer conditions. After a brief recovery, a second step occurs. At 12,700±52 year BP, δ*D*_aq_ values decrease again by at least 34‰. The drop in δ*D*_aq_ values straddles the pollen–stratigraphic AL–YD transition, which is a regional marker for major environmental changes resulted from hemispheric-scale cooling^27^ (Fig. 2). This shift coincides with a 26‰ rise in Δδ*D*_terr-aq_ and a ∼3 °C decrease in summer temperatures, indicating a further change towards drier and colder summer conditions.

In contrast, the synchronized NGRIP record shows that the first decline in δ*D*_aq_ values at HÄ coincides with rising δ^18^O values, corresponding to the warm Greenland Interstadial 1a (GI-1a). The local δ*D*_aq_ minima and Δδ*D*_terr-aq_ maxima at HÄ are synchronous with the start of the cold Greenland Stadial 1 (GS-1; 12,882±13 year BP; Fig. 2), defined in NGRIP ice cores as a rapid shift in *d*-excess^22^. Conversely, the second decline in δ*D*_aq_ values at HÄ occurs when NGRIP δ^18^O had already reached minimum values.

**Figure 3:**
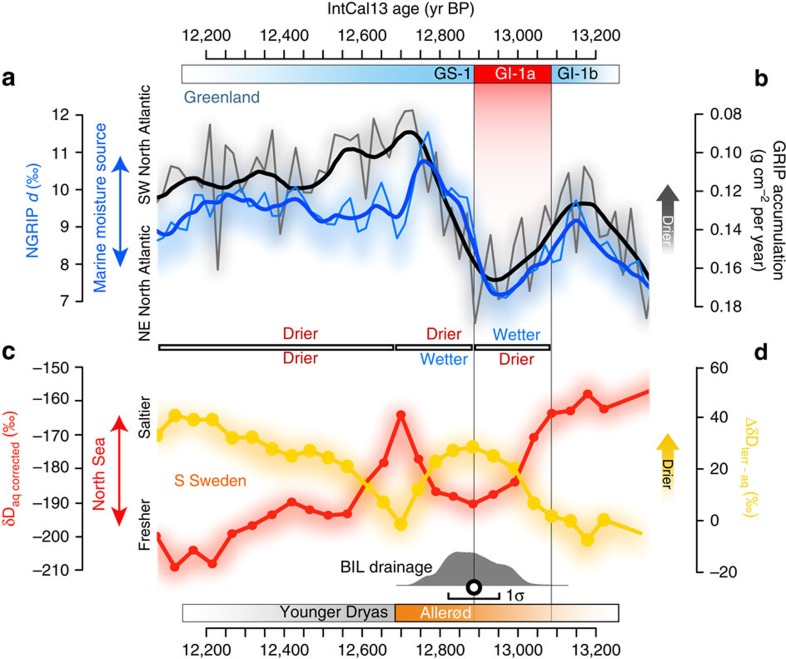
Comparison between Greenland and Hässeldala hydrological proxies at the onset of the YD pollen zone. Synchronized (**a**) NGRIP *d*-excess^22^ and (**b**) GRIP snow accumulation^23^ records compared to Hässeldala (**c**) δ*D*_aq_ corrected for ice volume, temperature and post-glacial isostatic uplift changes (δ*D*_corr_; Supplementary Methods), and (**d**) terrestrial evapotranspiration. The δ*D*_corr_ is a proxy for δ*D* of precipitation reflecting anomalies in distillation of the water vapour at the marine moisture source^25^, primarily driven by input of isotopically depleted freshwater. Note that the ∼19‰ decrease in δ*D*_corr_ (∼2.4‰ decrease in δ^18^O) during the late Allerød pollen zone is in agreement with a −2.5‰ shift in δ^18^O recorded in benthic foraminifera off the west coast of Southern Sweden^46^. The temporal evolution of the regional hydrological conditions is also displayed. Greenland records are presented at 20-year resolution with bold lines indicating the 60- year moving average. All records are presented with shadings indicating empirical 68% uncertainty bounds based on analytical and age-model errors. Vertical axes are oriented such that dry conditions plot upwards (note reverse axis for GRIP accumulation). Shown is also the combined probability of a number of calibrated radiocarbon dates constraining the age of the first drainage of the Baltic Ice Lake, inferred from deglaciation of the outlet in south-central Sweden and rapid isolation of lakes in the outlet area at Mt. Billingen (Supplementary Methods). 68% uncertainty (bar) and median age (circle) are also presented. Records are consistently displayed on the same IntCal13 time scale.

The comparison of HÄ δ*D*_corr_ and Δδ*D*_terr-aq_ records with NGRIP *d*-excess and GRIP accumulation rates shows two separate phases during GI-1a and during the first ∼180 years of GS-1 (Fig. 3). Each of these are characterized by a hydroclimate dipole across the eastern North Atlantic. GI-1a is marked by increasingly fresher North Sea surface conditions and inhibited moisture transport to HÄ. This interval coincides with a progressive north-eastward shift of the North Atlantic source of Greenland precipitation (more proximal) and with enhanced moisture transport to the summit^24^.

At the GI-1a/GS-1 transition, FIS meltwater discharge culminates and the hydroclimate dipole rapidly inverts its sign, marking the start of a brief hydroclimatic recovery, which lasted for ∼180 years. This ∼180-year-long interval, which represents a transitional phase between the onset of GS-1 in Greenland and North Hemispheric cooling^17^,^27^, appears to have been characterized by a temporary return to more saline conditions in the North Sea and stronger advection of moisture to HÄ. By contrast, the moisture source of Greenland precipitation moves south-westwards (more distant), resulting in less effective moisture transport to the summit^24^. This transition has been attributed to a southward diversion of the westerly winds and stronger zonal circulation owing to sea-ice expansion in the North Atlantic^17^. Consistent with modern observations^28^, stronger zonal winds can more efficiently route warm and saline North Atlantic waters to the North Sea, but also cause a south-westward shift of Greenland's precipitation source^24^.

After the ∼180-year-long transitional phase and coinciding with the regional AL–YD pollen-zone boundary, Greenland's hydroclimate stabilized to a stadial mode. The establishment of stadial conditions in Southern Sweden occurred, however, one century later when progressive freshening of surface waters in the North Sea caused a gradual drop in summer temperatures and precipitation (Figs 2 and 3). We interpret these asynchronous events as an expression of the southward migration of North Atlantic storm tracks^17^ coincident with a gradually more persistent summer sea-ice growth in the Nordic Seas^29^.

The succession of surface freshening/salification events inferred from HÄ records through GI-1a and GS-1 is in line with reconstructions from the Skattegat–Kattegat^30^, the North Sea^31^ and the Norwegian Sea^29^,^32^. We thus suggest that increasingly stronger melting of the FIS in response to the Late AL warming (Fig. 2) played a central role in the hydrological cycle of the eastern North Atlantic at the transition into the YD climatic reorganization.

### Climate model simulations

**Figure 4:**
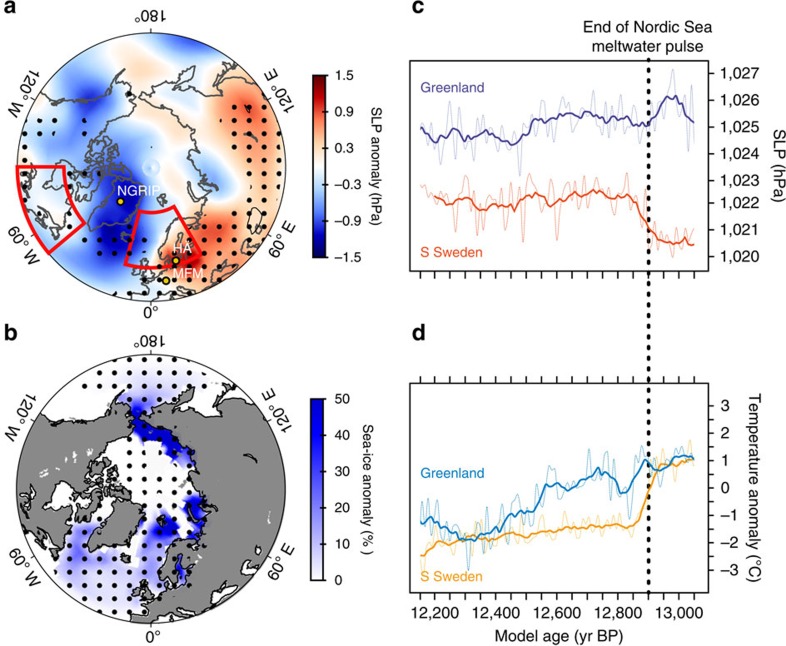
Modelled climate changes under Fennoscandian Ice Sheet freshwater forcing conditions. Summer changes (JJA) in (**a**) sea-level pressure and (**b**) sea-ice cover between the 50 years preceding the abrupt cooling (13,000–12,951 model year BP) and the abrupt transition period (12,940–12,891 model year BP). Significance levels are indicated by black stippling (95%). Sites referred to in the text are shown. The areas delimited in red show the location where freshwater forcing was prescribed. Time series of summer (**c**) sea-level pressure and (**d**) surface temperature decadal mean (light solid line) anomalies averaged over 57.5°–76.0° N and 20.0°–40.0° W domain for Greenland and 50.0°–65.0° N 5.0°–35.0° E domain for Sweden, respectively. The bold lines show a 50-year running mean. The dotted line represents the end of the modelled Nordic Sea meltwater pulse. Sea-level pressure anomaly changes were estimated after removing the related global mean.

**Figure 5:**
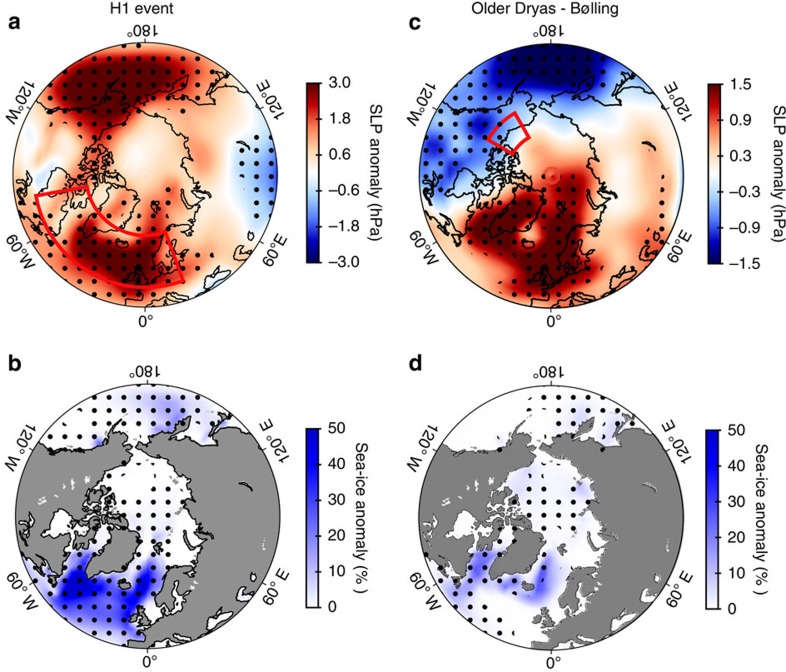
Modelled change in SLP and Sea Ice under Laurentide Ice Sheet freshwater forcing conditions. Summer changes (JJA) in (**a**) sea-level pressure and (**b**) sea-ice cover during Henrich Event 1 (H1), calculated comparing the Last Glacial Maximum mean state (19,050–19,000 model year BP) and the H1 climatology (17,050–17,000 model year BP). (**c**,**d**) Same as in **a** and **b** for the model counterpart of the Bølling-Older Dryas pollen–stratigraphic transition (14,250–14,200 minus 14,350–14,300 model year BP). Significance levels are indicated by black stippling (95%). The area delimited in red shows the region where meltwater forcing was prescribed in the model. Sea-level pressure anomaly changes were estimated after removing the related global mean.

To investigate our hypothesis, we turn to a transient simulation of the last 21,000 years performed with a coupled atmosphere–ocean climate model^33^ (Methods). The model shows great sensitivity of regional climate to a relatively weak FIS freshwater pulse (0.011 Sv) in the Nordic Seas during the Late AL. The freshwater forcing generates a summer sea-level pressure (SLP) dipole across the North Atlantic with deeper Icelandic low pressure and higher SLP over Northern Europe relative to the preceding phase (Fig. 4). The SLP dipole is a distinct feature in the model and is associated with FIS meltwater forcing only as it is absent when freshwater is discharged from North American sources (Fig. 5). The increased SLP, the surface cooling and the increased sea-ice cover simulated in the Norwegian and Barents Seas (Fig. 4) support the ∼2 °C decline in summer temperatures and progressively drier conditions recorded at HÄ during GI-1a (Fig. 2). These results are consistent with evidence of cooling recorded in other North European records during the same period^27^. Furthermore, a deeper Icelandic low pressure suggests a closer moisture source for Greenland precipitation, which is consistent with the δ^18^O enrichment in Greenland ice cores during GI-1a. The model output are further supported by high-resolution δ*D* records from Meerfelder Maar (MFM) in Western Europe^17^, where relatively wetter conditions are inferred during GI-1a, in contrast to drier conditions in Northern Europe (Supplementary Discussion; Supplementary Fig. 4).

## Discussion

In light of our results we argue that persistent FIS ice-mass loss at the end of the AL interstadial and the resulting freshening along the continental shelf of the Nordic Seas, which resulted in an early stage of cooling in Northern Europe, have likely determined the timing of hydrological shifts in Greenland at the GI-1a/GS-1 transition. Analogously to mechanisms invoked for the onset of cold climatic phases during the last glacial cycle^9^,^10^ and the present interglacial^34^, we posit that when sea ice reached a critical extent in the North Sea, Norwegian and Barents Seas, the excess of sea ice was transported to the subpolar North Atlantic via oceanic recirculation in the Nordic Seas. Potentially a sudden westward drainage of the Baltic Ice Lake through the south-central Swedish lowlands, as suggested by the available chronological evidence relating to deglaciation of the spillway (Figs 1 and 3; Supplementary Discussion; Supplementary Methods; Supplementary Fig. 9; Supplementary Table 2), may have contributed to drive sea ice and freshwater to the western sector of the Nordic Seas. Recirculation of sea ice in the Nordic Seas could have delivered ice to the subpolar North Atlantic to locations beyond the limits expected from local climatological conditions. The displacement of sea ice would have then caused the aforementioned southward shift of North Atlantic storm tracks at the onset of GS-1 and large-scale colder conditions. The model simulations support this interpretation. High-pressure anomalies over mid-to-high latitudes take place together with a westward and southward migration of sea ice in the North Atlantic (Fig. 5). In contrast, the SLP dipole pattern occurs only when sea-ice growth is confined to the eastern sector of the Nordic Seas (Fig. 4; Supplementary Figs 10 and 11). However, further studies are required to conclusively attribute a rapid export of sea-ice excess into the subpolar North Atlantic to a nonlinear behaviour of sea-ice growth in the eastern Nordic Seas or to a catastrophic meltwater discharge.

In conclusion, we provide a plausible mechanism for the linkages between FIS freshwater inputs to the Nordic Seas and North Atlantic hydroclimate patterns, reconciling the timing of freshwater forcing with the major isotopic excursions recorded in Greenland ice cores at the end of the Last Termination. Altogether, we suggest a new coherent concept for the inception of GS-1 and ultimately the YD, which may be critical to gauge future climate simulations.

## Methods

### Chronology

The chronology was established using a composite Bayesian age model based on 49 AMS ^14^C dates from terrestrial plant macrofossils. 21 ^14^C dates were transferred from a previously studied core^35^ by correlating total organic content records via a Monte Carlo alignment method^12^. At HÄ, core correlation is facilitated by the small size of the basin, which extends over an area of approximately 20 m^2^, resulting in total organic content records from adjacent cores to exhibit the same high resolution and identifiable lithostratigraphic patterns (for example, ref. 12).

### Lipid biomarker analysis

Fifty-four freeze-dried samples (∼8 cm^3^) were extracted from the sediments via sonication with dichloromethane: methanol (9:1) for 20 min; and subsequent centrifugation. This was repeated three times and supernatants were combined. Aliphatic hydrocarbon fractions were isolated from the total lipid extract using silica gel columns (5% deactivated) that were eluted with pure hexane. The saturated hydrocarbon fraction was separated by desulphurization over 10% AgNO_3_–SiO_2_ silica gel using pure hexane as eluent. Saturated hydrocarbon fractions were analysed by gas chromatography—mass spectrometry for identification and quantification, using a Shimadzu GCMS-QP2010 Ultra. Isotope ratios were determined using a Thermo Finnigan Delta XL mass spectrometer and all analyses were performed in triplicate. A standard mixture of *n*-alkanes with known δ*D* composition (mix A4, provided by A. Schimmelmann, Indiana University, USA) was run several times daily to calibrate the CO_2_ reference gas used for conversion of δ*D* values to VSMOW scale.

### Chironomid analysis

Samples for chironomid analysis of the HÄ sequence were taken every 2 cm. The aim was to obtain over 100 head capsules for each sample. Studies have demonstrated that 50 head capsules are an adequate minimum to establish species diversity in a sample and to provide reliable temperature estimates^36^,^37^. In most samples, 1–2 g of sediment was sufficient to obtain over 100 head capsules. The chironomid larval head capsules were prepared for identification following the procedure in Brooks *et al*.^38^ The head capsules were identified using a compound microscope at × 100 to × 400 magnification, with reference to Cranston^39^, Wiederholm^40^, Rieradevall and Brooks^41^ and Brooks *et al*.^42^ A modern Norwegian temperature calibration data set was used to derive the chironomid–inferred temperatures (ref. 43, and unpublished). Root-mean-squared-error of prediction (RMSEP) of the 2-component WA-PLS inference model was 1.12 °C, the coefficient of determination (r^2^) was 0.92 and the maximum bias was 0.77 °C.

### Model description

We analysed the simulation of the Transient Climate of the last 21 kyr (refs 33, 44) (TraCE-21ka). To perform this experiment the National Center for Atmospheric Research (NCAR) Community Climate System Model 3 (CCSM3) has been used. The atmospheric model is the Community Atmospheric Model 3 with 3.75° × 3.75° horizontal resolution and 26 hybrid vertical levels. The ocean model is the NCAR implementation of the Parallel Ocean Program with 25 vertical levels. The longitudinal resolution of the ocean model is 3.6° and the latitudinal resolution is variable, with finer resolution near the equator (∼0.9°). The model is coupled to a dynamic global vegetation module. The model is forced by realistic insolation, atmospheric CO_2_, continental ice sheets and meltwater discharge as described in details in Liu *et al*.^33^ (Supplementary Table 3). The model is able to consistently replicate many major features of the deglacial surface temperature evolution in agreement with reconstructions from various proxy records over the globe. The model reproduces the Northern Hemisphere cooling from the LGM into Heinrich 1 event, the abrupt warming into the Bølling–Allerød warm periods, the cooling into the Younger Dryas, and hence the following recovery to the warm climate into the Holocene^33^.

## Additional information

**How to cite this article:** Muschitiello, F. *et al*. Fennoscandian freshwater control on Greenland hydroclimate shifts at the onset of the Younger Dryas. *Nat. Commun.* 6:8939 doi: 10.1038/ncomms9939 (2015).

## Supplementary Material

Supplementary Information Supplementary Figures 1-11, Supplementary Tables 1-3, Supplementary Discussion, Supplementary Methods and Supplementary References.

Supplementary Data age-model output; chironomid-based summer temperature data; hydrogen isotope ratios form lipid biomarkers
