# Supplementary material for: Fennoscandian freshwater control on Greenland hydroclimate shifts at the onset of the Younger Dryas
Source: Nat Commun. 2015 Nov 17;6:8939. doi: 10.1038/ncomms9939 (PMC4660357; doi:10.1038/ncomms9939)
Supplement: Supplementary Information — Supplementary Figures 1-11, Supplementary Tables 1-3, Supplementary Discussion, Supplementary Methods and Supplementary References. [file ncomms9939-s1.pdf]

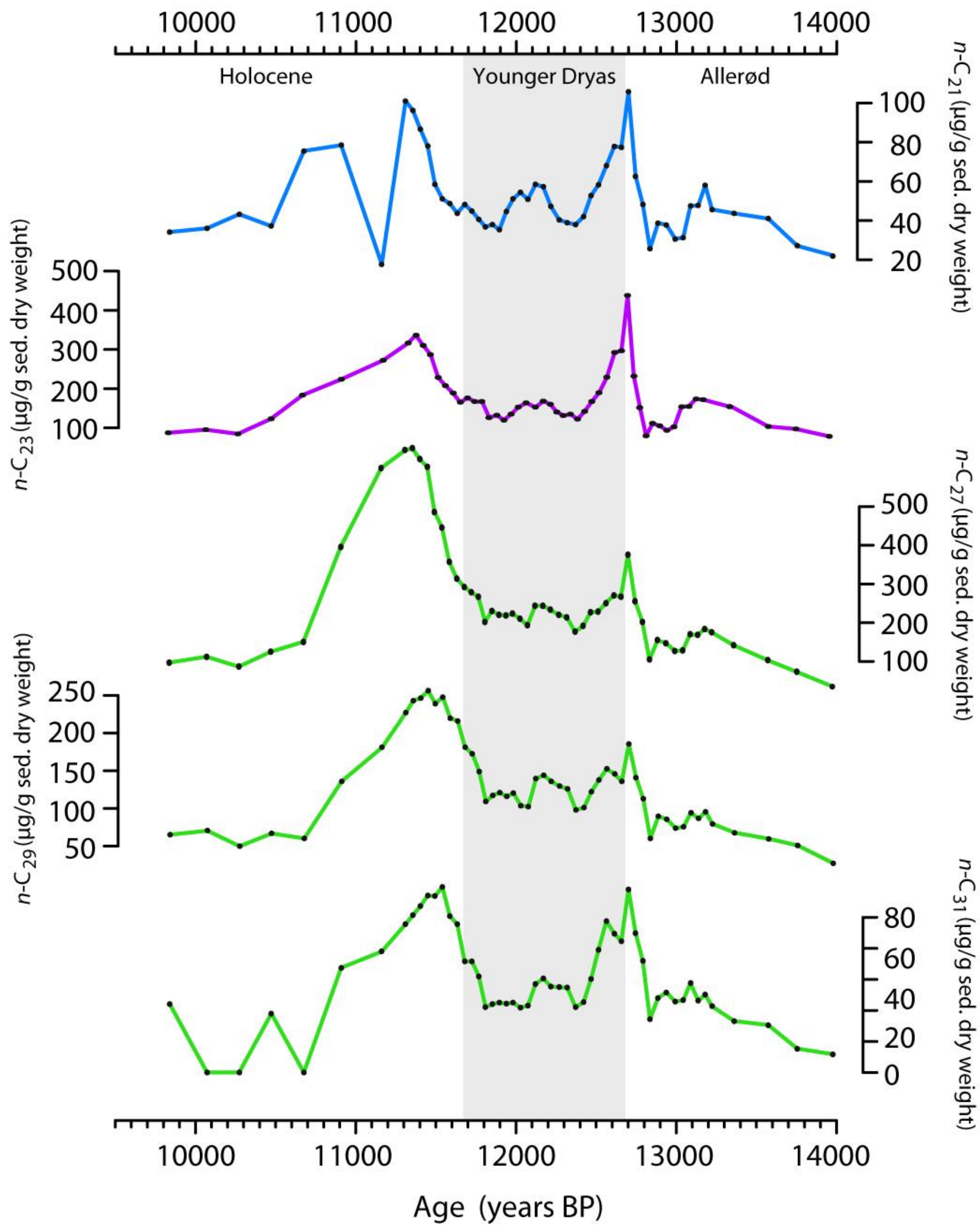

Supplementary Figure 1. Abundance of *n*-C_{21}, *n*-C_{21}, *n*-C_{27}, *n*-C_{29}, *n*-C_{31} alkanes present in sediments from Hässeldala. Major local pollen zone boundaries are also shown for reference. All *n*-alkane concentrations increase substantially before the onset and the termination of the Younger Dryas, respectively.

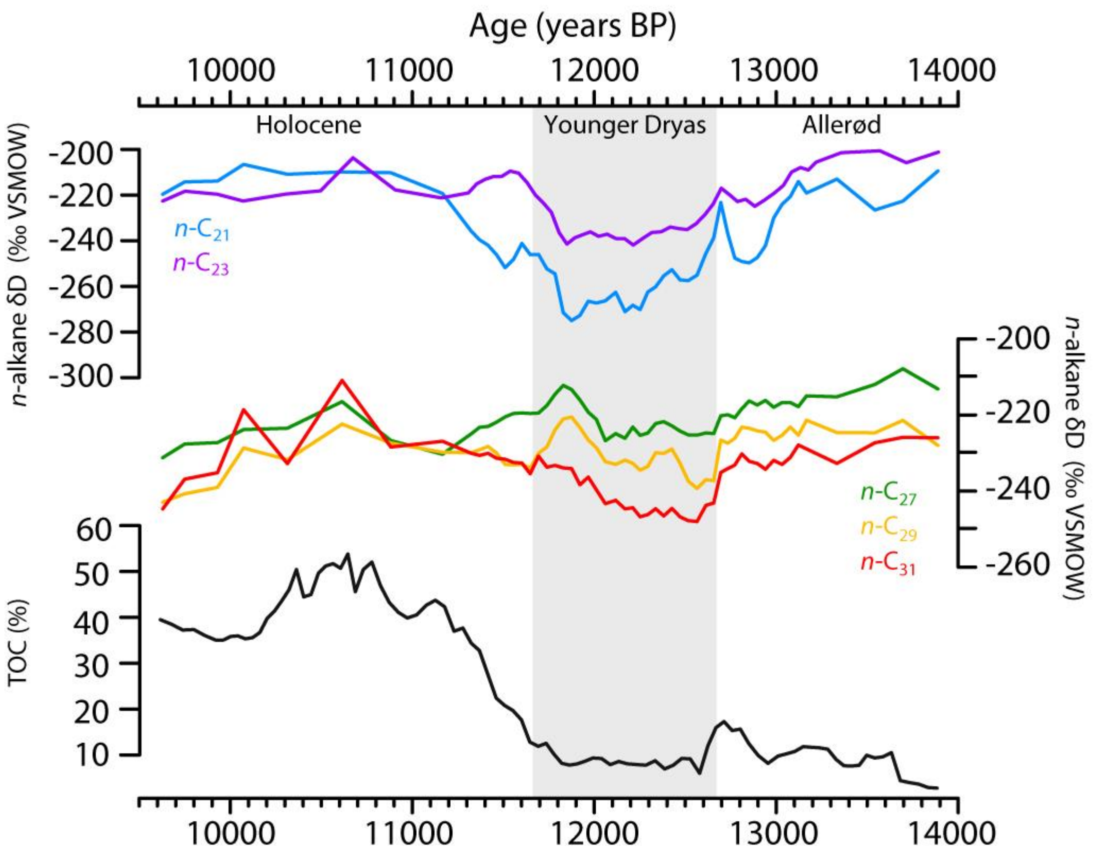

Supplementary Figure 2. Mean δD values of *n*-alkanes present in sediments from Hässeldala. Major local pollen zone boundaries and the TOC record are also shown for reference.

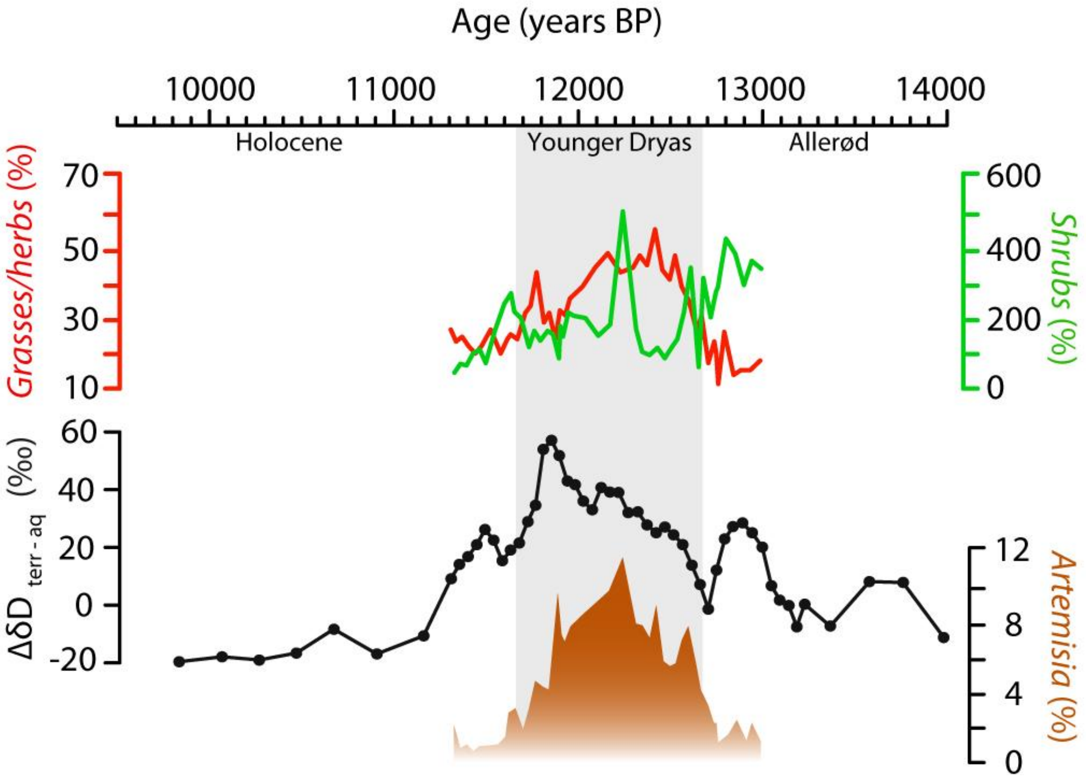

Supplementary Figure 3. Evapotranspiration and vegetation change records. Comparison of mean $\Delta\delta D_{terr\text{-}aq}$, a proxy for soil evapotranspiration, and pollen percentages for total grasses/herbs, total shrubs and *Artemisia* (a plant taxon adapted to dry conditions) in Hässeldala sediments.

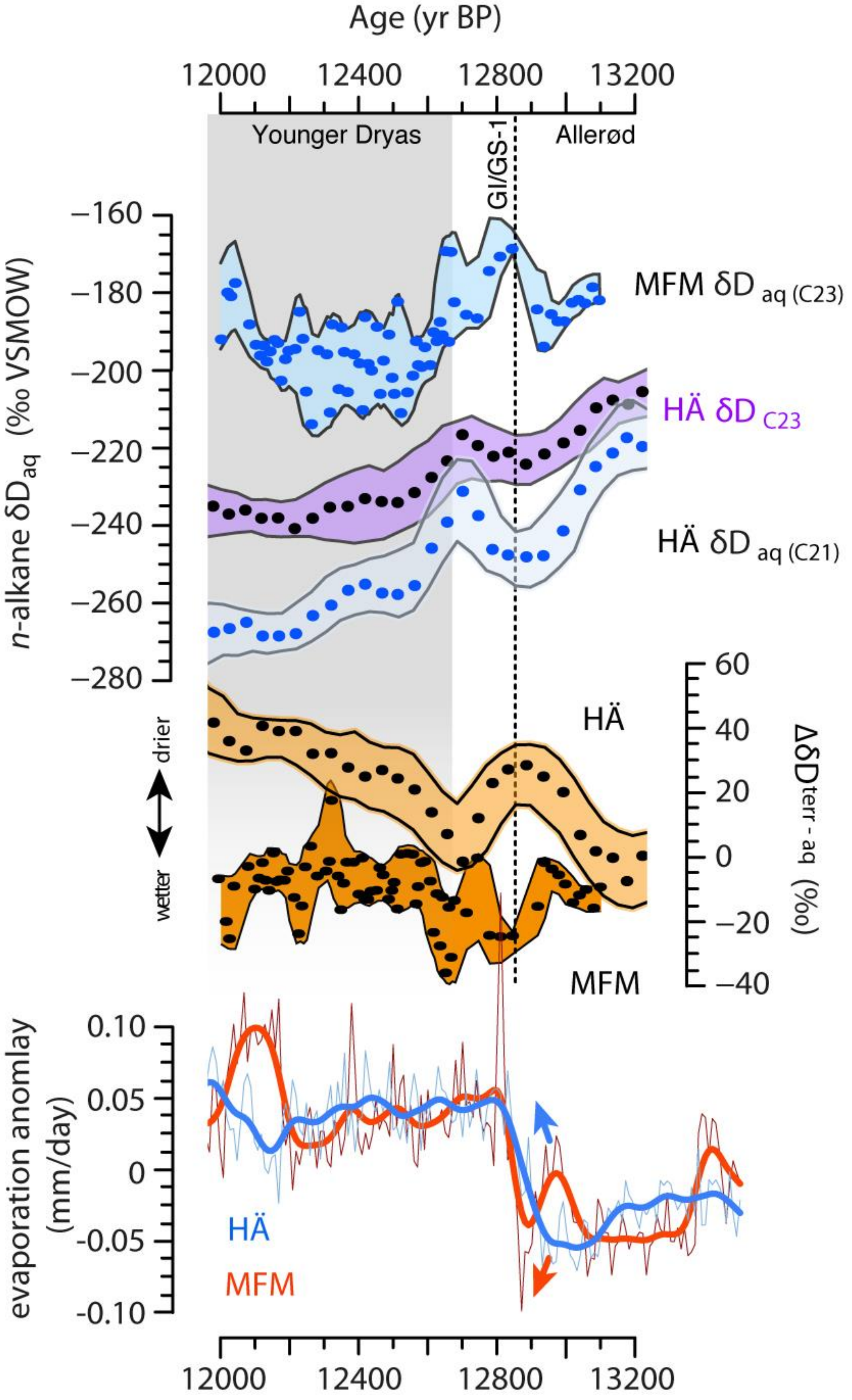

Age (yr BP)
12000
12400
12800
13200
Younger Dryas
GI/GS-1
Allerød
−160
−180
−200
−220
−240
−260
−280
n-alkane δDaq (‰ VSMOW)
MFM δD aq (C23)
HÄ δD C23
HÄ δD aq (C21)
60
40
20
0
−20
−40
ΔδDterr - aq (‰)
HÄ
MFM
drier
wetter
0.10
0.05
0
-0.05
-0.10
evaporation anomlay (mm/day)
HÄ
MFM
12000
12400
12800
13200

Supplementary Figure 4. Comparison of δD_{aq} and Δδ$D_{terr\text{-}aq}$ records from Meerfeleder Maar and Hässeldala sediments. The δD_{aq} in Meerfelder Maar is associated with the δD composition of *n*-C_{23} alkanes, whereas in Hässeldala it is associated with the δD composition of *n*-C_{21} alkanes. The δD composition of *n*-C_{23} alkanes from Hässeldala's sediments is also shown. All records are presented with shadings indicating empirical 95% uncertainty bounds based on analytical and age-model errors. The onset of the local pollen-defined Younger Dryas at the sites and the start of Greenland Stadial 1 in are also shown. The records are displayed together with the local soil evaporation anomaly as modeled in the TraCE-21ka simulations. Note evaporation anomalies of opposite signs at the end of the meltwater forcing in the Nordic Seas and consistent with the reconstructions.

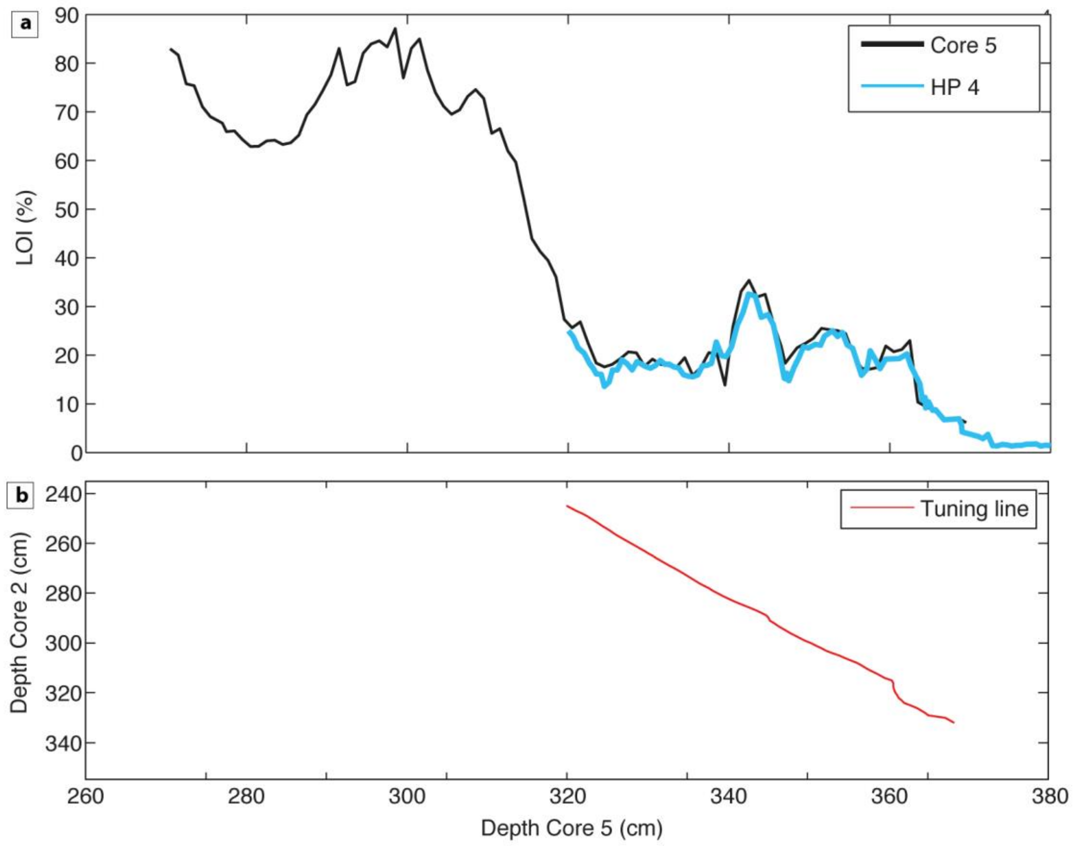

Supplementary Figure 5. Sediment core alignment. Results of the Monte Carlo alignment of LOI data between core HP4 and Core 5. **a** LOI data of HP4 and Core 5 plotted on the depth scale of Core 5 after the alignment. **b** Optimal tuning line that relates the depth scales of the two records.

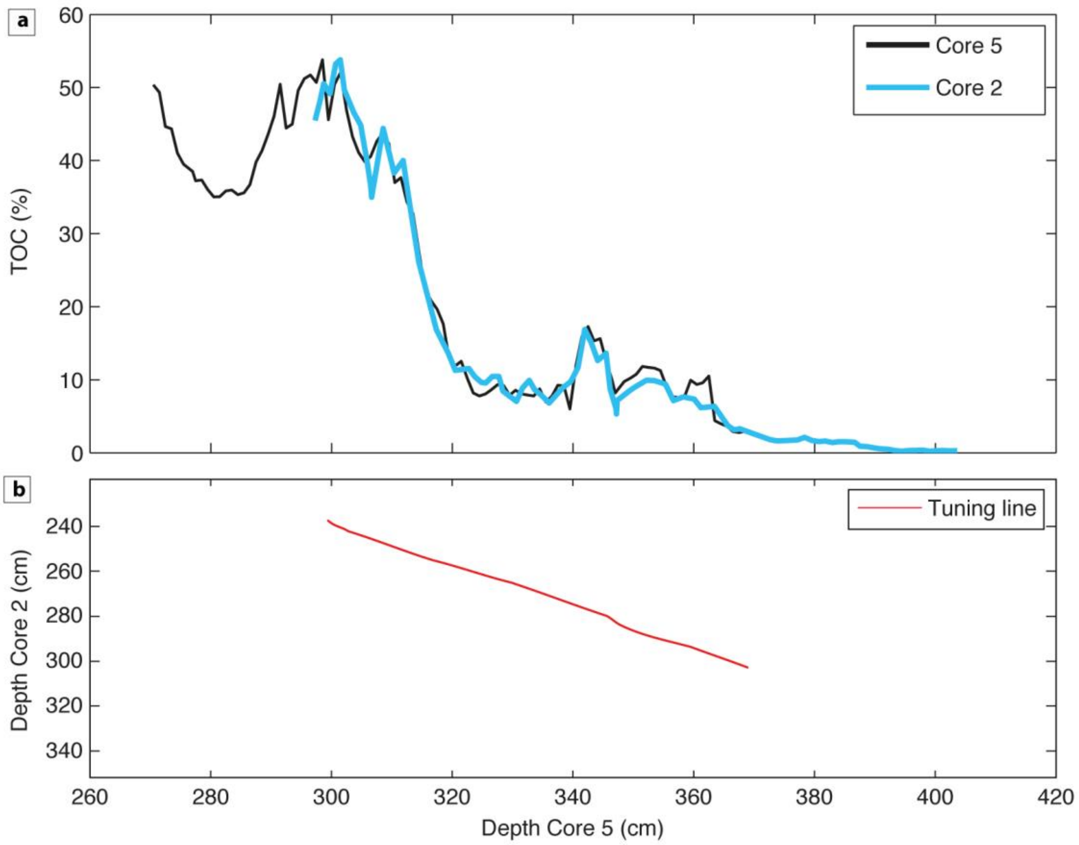

Supplementary Figure 6. Sediment core alignment. Same as in Supplementary Figure 5 for Monte Carlo alignment between Core 2 and Core 5 using TOC data.

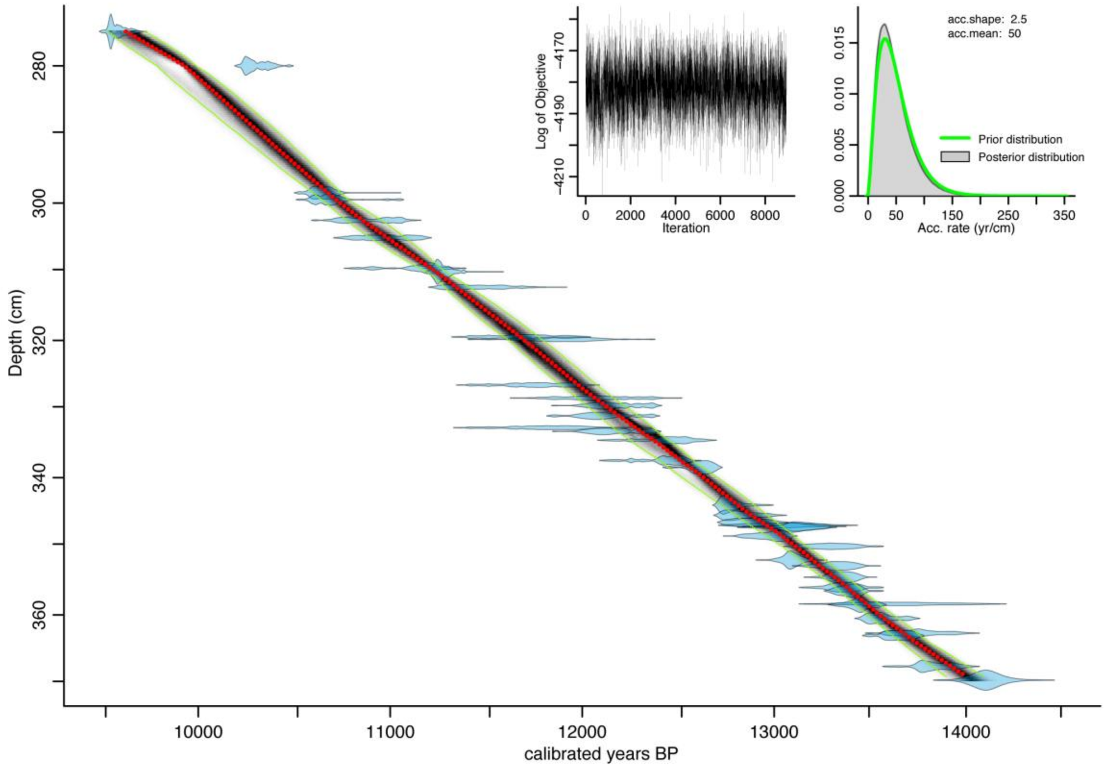

Supplementary Figure 7. Bayesian age-depth model. Age model of Hässeldala's composite radiocarbon-dated sequence based on the Bayesian analysis procedure of Bacon2.2 (ref. 1), using the IntCal13 calibration curve (ref. 2). In the graph calibrated ^{14}C dates are displayed in cyan and the modelled age-depth relationship is shown. Darker shading indicates more likely calendar ages, green lines show 95% confidence envelopes, and the red dotted line shows the weighted mean age for each depth. Upper right panels depict the MCMC iterations and prior-posterior distribution for the accumulation rate.

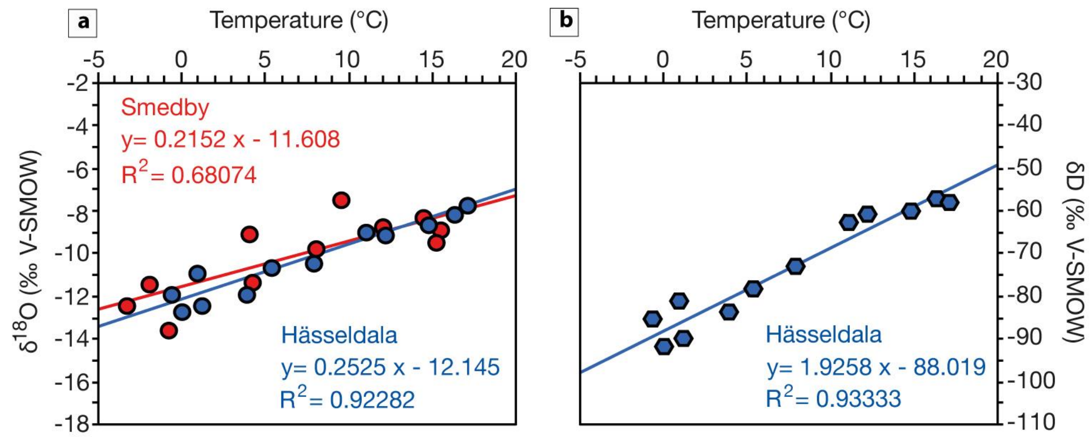

Supplementary Figure 8. Correlation between climatological monthly mean δ precipitation values and air temperature at Hässeldala. Data from the Online Isotopes in Precipitation Calculator (OIPC) (ref. 3) and Era-Interim. **a** Annual $\delta^{18}O$-temperature relationship at Hässeldala (blue) and at Smedby (red), the closest meteorological station with both observations of δ values in precipitations (i.e. $\delta^{18}O$) and air temperature. Observational data from Smedby are here used to test the goodness of the model-based data used to construct (**b**) the local annual δD-temperature relation.

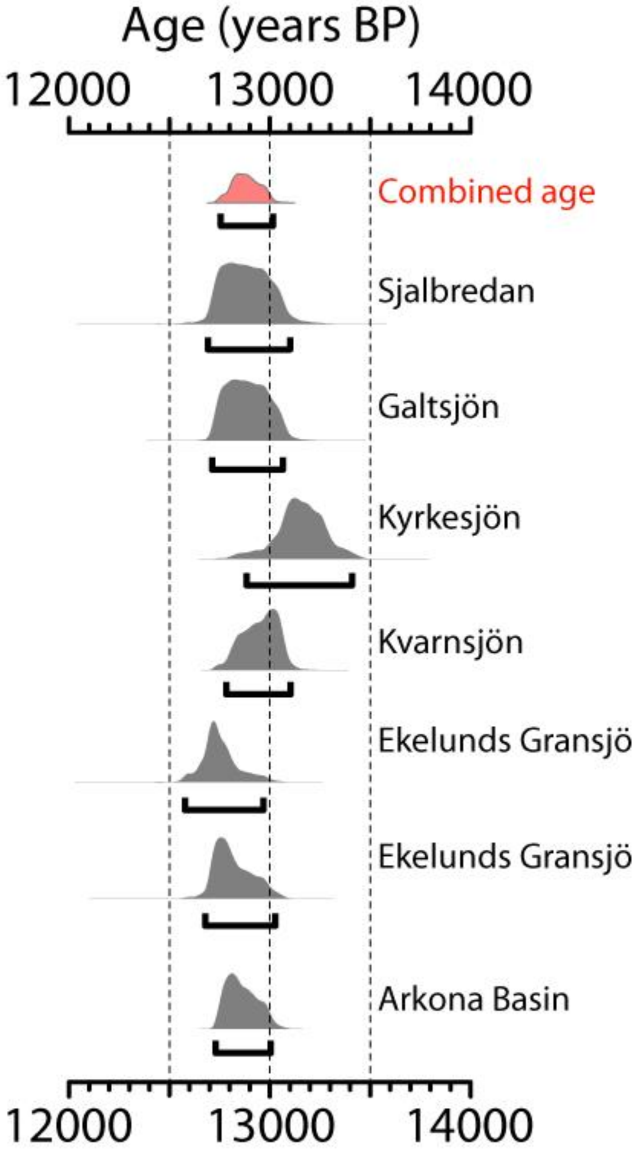

Supplementary Figure 9. Combined probability of published AMS ^{14}C dates constraining the age of the first drainage of the Baltic Ice Lake. The combination was made using the 'R-combine' function in OxCal4.2 (ref. 4). For reference the dates are also displayed as single events.

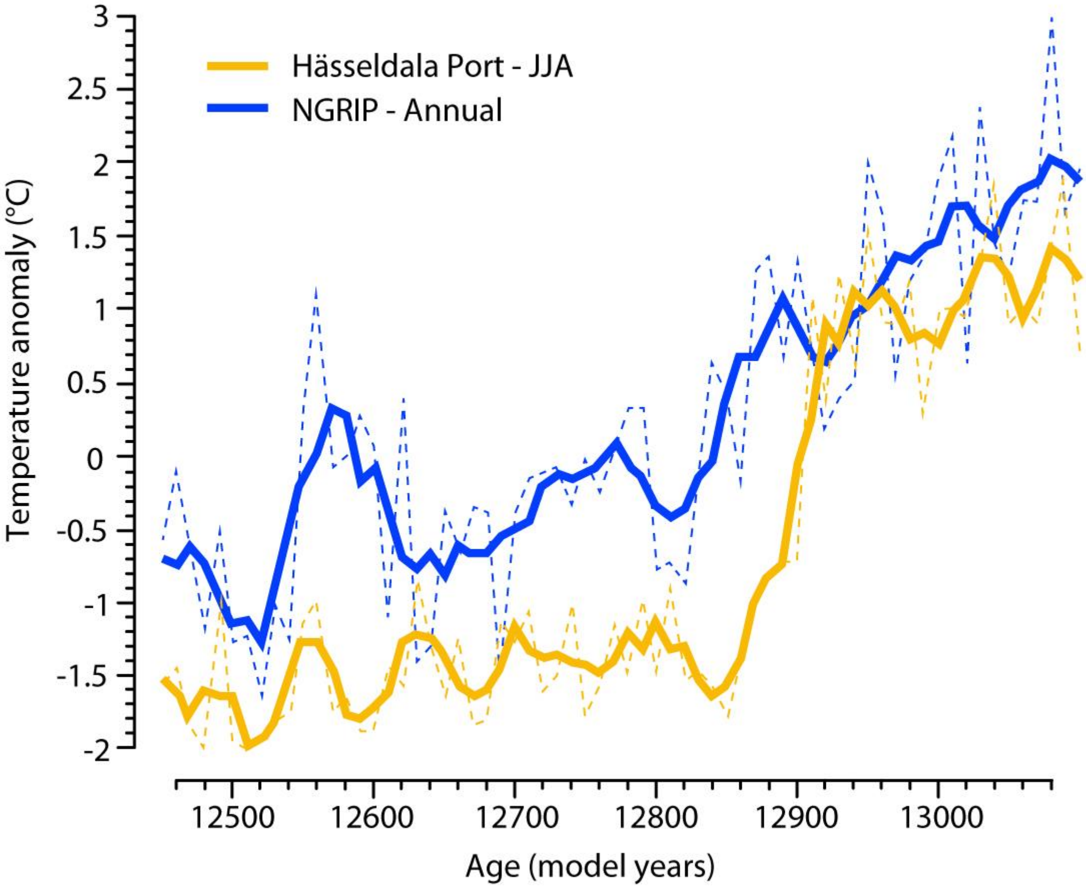

Supplementary Figure 10. Decadal summer (JJA) and annual surface air temperature anomalies for Hässeldala (dashed yellow line) and NGRIP (blue dashed line) location, respectively. 30-year running average windows are also shown (bold lines).

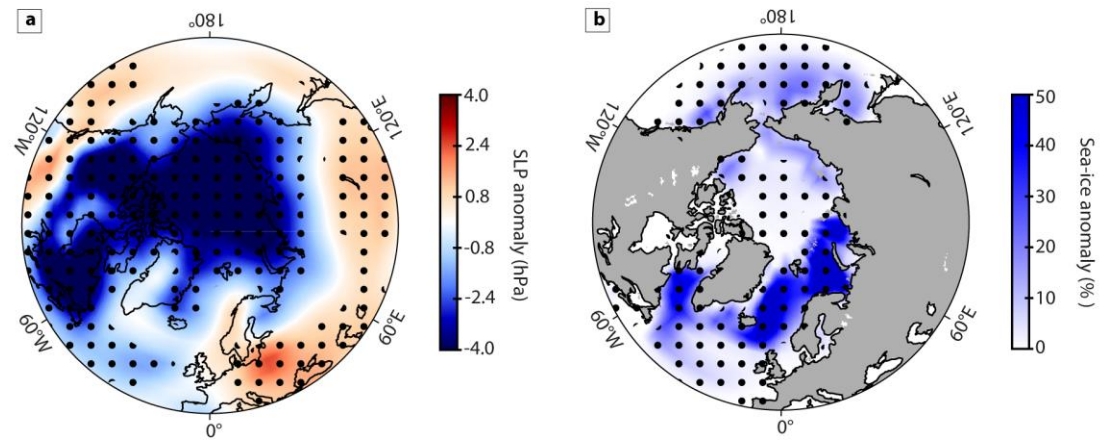

Supplementary Figure 11. Summer changes (JJA) in (**a**) sea-level pressure and (**b**) sea-ice cover between a reference period without or very little meltwater forcing (14350-14000 model yr BP) and the abrupt cooling period associated with meltwater forcing in the Nordic Seas (12940-12891 model yr BP). Significance levels are indicated by black stippling (95%).

Supplementary Table 1 – AMS ^{14}C dates for Hässeldala based on selected terrestrial plant remains and used to construct the composite age-depth model. Blue samples refer to dates transferred from Core 2 to Core 5.

Sample depth (cm)	Thickness error (cm)	Sample ID	Material analysed	^{14}C age (yr)	^{14}C error (1 sigma)	Used in the age model
369.5	0.5	UBA-20296	B, D	12211	57	Yes
367.5	0.5	UBA-20297	D, S	11972	62	Yes
366.6	1.15	Ua-20516	B, D, T	12355	190	No
365.25	0.75	UBA-20298	B, D, S	11673	56	No
363	0.5	UBA-20299	B, D	11819	57	Yes
362.65	1.4	Ua-20517	B, D	11920	90	Yes
360.5	0.5	UBA-20300	B, D	11735	60	Yes
360.15	0.75	Ua-20518	B, D	11990	110	No
358.5	0.5	UBA-20301	B, D	11614	55	Yes
358.4	0.95	Ua-20519	B	11805	240	Yes
356.5	0.5	UBA-20302	B, D	11565	56	Yes
356	1.1	Ua-20520	B	11525	85	Yes
354.5	0.5	UBA-20303	B	11515	65	Yes
352.85	1.2	Ua-20521	T	11490	85	Yes
352	1	UBA-20304	B	11225	60	Yes
350	1.25	Ua-20522	B	11455	125	Yes
348.5	0.5	UBA-20305	B	11118	60	Yes
347.25	1.2	Ua-20523	B, T	11245	95	Yes
347.1	0.9	Ua-20524	B, T	11275	95	Yes
346.95	1.05	Ua-20525	B	11200	165	Yes
346.5	0.5	UBA-20306	B	10998	63	Yes
345.5	1	Ua-20526	B, T	10935	80	Yes
344	1	UBA-20307	B	10894	63	Yes
339.9	1.8	Ua-20527	B, T	11070	135	No

338.5	0.5	UBA-20308	B	10644	58	Yes
337.5	0.5	UBA-20309	B	10484	50	Yes
337.4	0.95	Ua-20528	B, T	10935	80	No
334.55	0.95	Ua-20529	B, T	10515	75	Yes
333.25	1.75	UBA-20310	B	10331	51	Yes
332.7	1	Ua-16740	B	10165	95	Yes
331.6	1.5	UBA-20311	B	10301	57	Yes
329.5	0.5	UBA-23306	Bi	10331	53	Yes
328.5	0.5	UBA-20312	B	10639	51	No
328.4	1.05	Ua-16745	B, D	10285	95	Yes
326.5	0.5	UBA-20313	B	10130	69	Yes
323.35	1.15	Ua-16747	B	9860	85	No
319.85	1.15	Ua-16750	B, C	10205	85	Yes
319.5	0.5	UBA-21574	B	10102	67	Yes
315.9	1.1	Ua-16752	B, BP, P	9720	90	No
312.25	1.1	Ua-16761	B, T	9955	90	Yes
310	1	UBA-22308	Bi, M	9864	47	Yes
309.5	1.15	Ua-16766	P	9765	85	Yes
305.05	1.15	Ua-16768	P	9625	70	Yes
302.5	0.5	UBA-21575	B	9532	59	Yes
299.5	0.5	UBA-21577	B	9442	52	Yes
298.5	0.5	UBA-21578	B	9416	47	Yes
290	1	UBA-21576	B, C	7496	55	No
280	1	UBA-23309	Bi	9129	50	Yes
275	0.5	UBA-21579	T	8605	46	Yes

B: *Betula nana* – leaves, leaf fragments, seeds, buds, twigs

Bi: Birch – twigs, leaf fragments, seeds, bark

BP: *Betula pubescens* - seeds

C: charcoal
D: *Dryas octopetala* – leaves, leaf fragments, seeds, buds
M: moss
P: Pine – needles
T: undetermined terrestrial material

Supplementary Table 2 – Published AMS ^{14}C dates constraining the age of the first drainage of the Baltic Ice Lake. Ages refer to lake isolations from the sea, which indicate timing of deglaciation west of the drainage outlet area at Mt. Billingen,near 11,000 radiocarbon years BP. Two dates refers to isolation owing to concomitant lowering of the Baltic Ice Lake in Blekinge. Dates were combined using the 'R-combine' function in OxCal4.2 (ref. 4) after calibration with the IntCal13 calibration curve (ref. 2). Only ages considered reliable by the original study authors and directly related to lake isolations were used. The temporal correspondence between the supposed first drainage of the Baltic Ice Lake and the regional pollen stratigraphy is well constrained and thus offers an indirect time marker in other regional sedimentary sequences (see text). ^{14}C dates from other sequences used to constrain timing of deglaciation at Mt. Billingen through pollen stratigraphic evidence were listed and noted with the entry "Pollen time marker". One ^{14}C date constraining the timing of the Baltic Ice Lake water-level fall is also listed.

Site	Location	^{14}C age (yr)	^{14}C error (1 sigma)	Material analysed	Used in this study	Reference	Original study interpretation
Galtsjön	Blekinge	11000	110	Bulk	Yes	Ref. 5	Isolation
Sjalbredan	Blekinge	10990	135	Bulk	Yes	Ref. 5	Isolation
Lake Bjärsjön	Mt. Billingen	10830	100	Bulk	No	Ref. 6	Pollen time marker
Lake Madtjärn	Mt. Kroppefjäll	10995	75	Tpm	No	Ref. 7	Pollen time marker
Domsjön	Hunneberg	11820	130	Bulk	No	Ref. 8	Spurious
Kyrkesjön	Hunneberg	11290	120	Bulk	Yes	Ref. 9	Isolation
Kyrkesjön	Hunneberg	10770	105	Bulk	No	Ref. 9	Spurious
Kvarnsjön	Hunneberg	11110	75	Bulk	Yes	Ref. 9	Isolation
Kroppsjön	Hunneberg	11870	110	Bulk	No	Ref. 9	Marine material
Kroppsjön	Hunneberg	11820	110	Bulk	No	Ref. 9	Marine material
Kroppsjön	Hunneberg	11610	110	Bulk	No	Ref. 9	Marine material
Kroppsjön	Hunneberg	11660	110	Bulk	No	Ref. 9	Marine material
Kroppsjön	Hunneberg	11390	105	Bulk	No	Ref. 9	Spurious
Ekelunds Gransjö	Hunneberg	10830	100	Bulk	Yes	Ref. 9	Isolation
Ekelunds Gransjö	Hunneberg	10900	100	Bulk	Yes	Ref. 9	Isolation
Ekelunds Gransjö	Hunneberg	11800	110	Bulk	No	Ref. 9	Marine material
Ekelunds Gransjö	Hunneberg	11670	90	Bulk	No	Ref. 9	Marine material
Alsjön	Hunneberg	10790	100	Bulk	No	Ref. 9	Spurious
Svartevattnet	Hunneberg	10730	100	Bulk	No	Ref. 9	Spurious
Core 258000	Arkona Basin	10980	55	Tpm	Yes	Ref. 10	Lake-level fall

Tpm: terrestrial plant macrofossil

Supplementary Table 3 – Pre-Younger Dryas meltwater fluxes in the TraCE experiment.

Age (yr)	NHMW (m kyr^{-1})	NHMW location	SHMW (m yr^{-1})	SHMW location
13870-12900	1	Nordic Sea	5	Ross Sea
13870-13400	4	Gulf of Mexico	/	/
13400-12900	2.4	Gulf of Mexico	/	/
13400-12900	1.6	St. Lawrence River	/	/

NHMW: Northern Hemisphere meltwater
SHMW: Southern Hemisphere meltwater

Supplementary Discussion

Sources of *n*-alkanes in sediments from Hässeldala Lake and suitability of δD records for hydrological investigations

In order to use the δD composition of sedimentary *n*-alkanes as a proxy for hydroclimatic conditions (e.g. evapotranspiration), it is important to first assess the possible aquatic versus terrestrial origin of these compounds in their specific lake environment[57]. In our study we employed the δD composition of *n*-C_{21}, and *n*-C_{27}, *n*-C_{29}, *n*-C_{31} alkanes as representative components of aquatic and terrestrial sources, respectively. The vegetation source attribution was established via comparison to local pollenstratigraphic records[58] and vegetation reconstructions for Blekinge[5, 13, 14]. The local pollen stratigraphy established for Hässeldala compares excellently with other local pollen stratigraphies established in the region and with the regional pollenstratigraphic framework for southern Sweden[14]. The amount of long-distance transported pollen (such as *Pinus*) or reworked pollen (such as deciduous tree pollen) is minor and the majority of the pollen encountered at Hässeldala reflects the local and/or regional vegetation pattern of shrubs and grasses/herbs. An unpublished macrofossil diagram for Hässeldala shows that shrubs, herbs and grasses (*Dryas octopetala*, *Salix polaris*, *Salix herbaceae*, *Betula nana*) are present throughout the Allerød and the Younger Dryas pollen zones and that tree macro remains only appear at the Younger Dryas-Holocene boundary.

Short-to-mid-chain n-alkanes, like the *n*-C_{21} alkane, are established biomarkers for aquatic plants[15, 16, 17]. At Hässeldala, the *n*-C_{21} alkane can be related to the submerged macrophyte *Myriophyllum* sp., possibly with minor contributions from *Batrachium* sp. and *Potamogeton* sp. These plants were common limnotypes in the region and continuously present throughout the Late Glacial[13]. *Myriophyllum* sp., especially, was abundant in lakes in the region during the regional Allerød and Younger Dryas pollen zones[13]. The *n*-C_{21} alkane has been shown to associate with the occurrence of *Myriophyllum*, but also with *Batrachium* and *Potamogeton*[16]. Pollenstratigraphic investigations in lakes close to Hässeldala[13] show that *Myriophyllum* sp. pollen percentages increase markedly in two distinct phases corresponding to the transition into and out of the Younger Dryas, respectively, which agrees well with the changes in *n*-C_{21} concentration observed in Hässeldala sediments (Supplementary Fig. 1).

This interpretation is also confirmed by geochemical data. For instance, C/N ratios in Hässeldala sediments are rather constant during the Allerød, Younger Dryas and Early Holocene pollen zones, with values ranging ~13 (ref. 18). This suggests a stable input of aquatic macrophytes over time with sedimentary values typical for a mixture of *Myriophyllum* and *Potamogeton*[19]. Analogously, the bulk organic $\delta^{13}C$ stratigraphy shows constant values ranging at −17‰ (ref. 18), very similar to those reported elsewhere in association with sediments characterized by large input of *Myriophyllum*, together with *Batrachium* and *Potamogeton* (between -17.5‰ and -19.5‰; ref. 20). Although these aquatic plant types have been shown to produce

relatively larger amounts of *n*-C_{23} alkanes as compared to *n*-C_{21} (ref. 16), we preferred to only use *n*-C_{21} in order to minimize the potential bias from higher terrestrial plants, which also produce, to some extent, mid-chain *n*-alkanes.

Conversely, long-chain *n*-alkanes, like the *n*-C_{27}, *n*-C_{29} and *n*-C_{31} alkanes, are main constituents of leaf waxes of higher terrestrial plants[21]. In Hässeldala sediments, we assigned these compounds to a mixture of leaves from *Betula nana* and *Salix polaris*. The *n*-C_{27} is generally synthesized by these two plant species[22]. In fact, the pollenstratigraphy of the Hässeldala sedimentary sequence indicates a marked increase in *Betula* sp. and *Salix* sp. pollen percentages at the Allerød-Younger Dryas transition and during the early Holocene[12], which is in line with changes in the abundance of *n*-C_{27} alkanes (Supplementary Fig. 1). The *n*-C_{29} is a ubiquitous alkane in terrestrial plants, whereas the *n*-C_{31} alkane is a main constituent in grasses[22, 23]. In Hässeldala sediments, grass/herb pollen counts decrease during the Late Younger Dryas and Early Holocene pollen zones, which disagrees with the distribution of *n*-C_{31} alkanes in our records (Supplementary Fig. 1). Rather, we also attribute the origin of both *n*-C_{29} and *n*-C_{31} alkanes to *Betula nana* and *Salix polaris/herbaceae,* which show a marked covariance with their respective concentrations, with marked peaks in pollen counts at the Allerød-Younger Dryas and Younger Dryas-Holocene pollen zone transitions (Wohlfarth et al. 2006; Supplementary Fig. 1).

In summary, we argue that the short-chain and long-chain *n*-alkanes discussed here originate from separate aquatic and terrestrial sources, respectively. This is also supported by the lack of any significant correlation between the δD values of *n*-C_{21}

and n-C_{27}, n-C_{29}, n-C_{31} alkanes, which gives us confidence that the $\Delta\delta D_{long\text{-}short}$ represents the actual $\Delta\delta D_{terr\text{-}aq}$ (ref. 11).

It should be noted that the δD records of long-chain *n*-alkanes exhibit a lower variability than the δD records of short-chain *n*-alkanes (Supplementary Fig. 2) and that the $\Delta\delta D_{terr\text{-}aq}$ appears thus mainly driven by changes in δD_{aq}. We argue that the freshwater forcing in the North Sea caused marked declines in δD_{aq} values at Hässeldala during the Late Allerød and the Younger Dryas pollen zone, respectively, but also caused a hampered moisture flux from the near marine source. The freshwater forcing thus indirectly induces drought conditions at Hässeldala. Mechanistically, the δD_{terr} responds to changes in evapotranspiration with less negative values relating to greater evapotranspiration under dry atmospheric conditions. Therefore, in our record the expected variability in δD_{terr} predicted by negative shifts in δD_{aq} values is offset by a concurrent rise in evapotranspiration - and accordingly in $\Delta\delta D_{terr\text{-}aq}$ values.

The effect of evaporative deuterium enrichment owing to enhanced evapotranspiration is particularly pronounced in shrub waxes as compared to other functional groups such as trees or grasses[24] due to the drought tolerance and high water-use efficiency in shrubs (e.g. ref. 25). However, the apparent low variability of δD_{terr} relative to δD_{aq} could have also been caused, to some extent, by admixture of older organic material, which resulted in a longer temporal integration of the stable isotopic composition of the *n*-alkane pool (see further discussion below).

Nonetheless, we consider the effect of enhanced evapotranspiration as the most likely primary factor subduing the variability of δD_{terr} at Hässeldala. In fact, we observe that the overall pattern of the evapotranspiration history predicted by the $\Delta\delta D_{terr\text{-}aq}$ would be the same even if we used the δD composition of *n*-alkanes typical of emergent aquatic plants, such as *n*-C_{23} (Supplementary Fig. 2; ref. 26), instead of the terrestrial-dominated δD signal (i.e. *n*-C_{27}, *n*-C_{29}, *n*-C_{31}). This suggests that even plants less prone to strong evapotranspirative enrichment of leaf waters than shrubs have recorded the same (although smaller in magnitude) drought signal. We therefore propose that the $\Delta\delta D_{terr\text{-}aq}$ signal at Hässeldala can be used, at least qualitatively, to reconstruct the catchment evapotranspiration history.

Enhanced evapotranspiration during the Late Allerød and the Younger Dryas pollen zone, as inferred from the $\Delta\delta D_{terr\text{-}aq}$ record, is also broadly in line with evidence from taxonomic indicators for dry climatic conditions observed in a new high-resolution pollen record from Hässeldala sediments (Supplementary Fig. 3). Moreover, a phase characterized by drier conditions during the Late Allerød pollen zone was also identified in diatom records from the same sedimentary core[27].

Effect of vegetation and local hydrology on $\Delta\delta D_{terr\text{-}aq}$

The $\Delta\delta D_{terr\text{-}aq}$ values are directly related to the isotopic fractionation between plant source water and leaf wax lipids, which is referred to as net or apparent fractionation[74]. The net fractionation values for C3 grasses (no C4 plants were present in southern Sweden) are generally lower than those for C3 shrubs[24]. A new

high-resolution pollen record from Hässeldala sediments shows an increase in grass/herb pollen percentage and a decrease in shrub pollen percentage at the onset of the Younger Dryas pollen zone (Supplementary Fig. 3). The increase in grasses/herbs and decrease in shrubs would have resulted in more negative $\Delta\delta D_{terr-aq}$ values, working in the opposite direction of the shift observed in our $\Delta\delta D_{terr-aq}$ record. Therefore, if such vegetation changes affected our $\Delta\delta D_{terr-aq}$ reconstruction, the record should underestimate the increase in evapotranspiration during the Younger Dryas pollen zone. Furthermore, during the Late Allerød pollen zone we observe no significant change in the contribution of grasses and shrubs (Supplementary Fig. 3). This observation is corroborated by other independent local and regional vegetation reconstructions (e.g. ref. 5, 12, 14) and implies that changes in vegetation cover can be dismissed as a potential contributing factor to the shifts in δD_{terr} values recorded at Hässeldala shortly prior to the Younger Dryas pollen zone.

We also dismiss the possibility that the trend observed in the δD_{aq} record was caused by changes in net fractionation values due to a shift in the aquatic plant community producing *n*-C_{21} alkanes. Vegetation reconstructions from nearby lakes[13] indicate that the aquatic plant community remained generally unchanged throughout the Late Glacial. This is also supported by aquatic environmental studies from the same sedimentary core at Hässeldala[27, 29], which show no significant change in the aquatic habitat structure throughout the Late Allerød and the first half of Younger Dryas pollen zones.

Moreover, we can also dismiss the contribution of local hydrologic changes as contributing factors driving the δD_{aq} shifts at Hässeldala during this interval. For instance, under dry climatic conditions, such as during the Younger Dryas pollen zone (Fig. 2), evaporative deuterium enrichment on lake water δD may have occurred. However, this effect would have worked in the opposite direction as the one observed (δD depletion). Thus, if evaporation had played a role, it would mean that the signal recorded in both the δD_{aq} and $\Delta\delta D_{terr\text{-}aq}$ would underestimate the hydrological conditions. Similarly, this can be argued for the relatively dry phase that occurred during the Late Allerød pollen zone (Fig. 2), which has been identified in other independent proxy records from the same sedimentary core[27].

Age of terrestrial long-chain *n*-alkanes

One important consideration when interpreting isotopic records from terrestrial *n*-alkanes is the possibility of smoothing and/or a delay in the recorded signal caused by pre-aging on land and time needed for transport to and deposition in the lake basin. However, most of the plant waxes are expected to have arrived either directly after abrasion from the leaves, or simply from leaf litter in autumn. Earlier studies on lakes with small catchment areas have indicated that terrestrial *n*-alkanes, or other soil-derived compounds, typically have radiocarbon ages that are less than a few hundred years older than those of the sediments in which they are embedded (e.g. ref. 30, 31, 32).

Older leaf wax ages have been observed in areas with higher erosion rates where a large stock of ancient soil carbon and/or large watersheds contributes carbon inputs (e.g. ref. 33, 34). For smaller catchments the most viable explanation is that a small fraction of the total carbon pool has a significantly lower ^{14}C content, thereby exerting a large effect on the average ^{14}C content, while the vast majority has been deposited within a few years after biosynthesis. Under such a scenario, the stable isotopic composition of the total pool will remain relatively unaffected by admixture of older organic material, especially if their stable isotopic compositions are similar.

Hässeldala has a small catchment where transport time from the catchment to the basin is expected to have been short. Furthermore, from the age-depth relationship we observe no change in accumulation that could indicate relatively higher erosion rates in the catchment area during the period under study (Supplementary Fig. 7). The only potential indication of an increase in erosional input of older organic material from land occurs at ~12800 yr BP, when the total organic content and the abundance of *n*-alkanes rise (Supplementary Fig. 1, 2). This, however, takes place ~300 years after the δD_{aq} values start to decline and the $\Delta\delta D_{terr\text{-}aq}$ values start to increase (Supplementary Fig. 2). Therefore, we dismiss the contribution of admixing of older *n*-alkanes on δD_{terr} during Greenland Interstadial 1a.

In summary, we argue that the δD_{terr} signal is virtually synchronous with that of the aquatic lipids. Even if there has been some smoothing and attenuation of the δD_{terr}, this would not make any significant difference until the start of the regional Younger Dryas, as the δD_{terr} shows significant changes only after the onset of the Younger

Dryas (Supplementary Fig. 2). A shift of a few hundred years of the δD_{terr} record during the Younger Dryas and Holocene, together with a rise in amplitude to account for any smoothing that may have occurred, would also not change our conclusions on the late Allerød pollen zone hydroclimate shifts recorded at Hässeldala. This would also make the observed contrast between δD_{terr} and δD_{aq} even larger during the Younger Dryas.

In addition, we note that the δD_{terr} values generally change in phase with δD_{aq} and in particular at the regional Allerød-Younger Dryas pollen boundary (Supplementary Fig. 2), suggesting that the δD_{terr} record is in fact synchronous with the other proxy records from Hässeldala.

Comparison with δD records from Meerfelder Maar

By comparing our records with a recently published high-resolution isotope reconstruction from Meerfelder Maar, Western Germany[35], we can better decipher the European-scale regional hydroclimate expressions around the onset of Greenland Stadial 1 and the regional Younger Dryas pollen zone.

We observe that both the δD_{aq} records from Meerfelder Maar and Hässeldala show a consistent trend towards more negative values at the start of the Younger Dryas pollen zone, which is suggested to reflect a hemispheric-scale cooling, more negative δD values of the ocean source associated with changes in precipitation pathways and sea-ice expansion in the North Atlantic[35]. More interestingly, the records suggest an opposite hydroclimate behaviour between southern Sweden and western Germany

during the Late Allerød pollen zone/ Greenland Interstadial 1a, with relatively higher δD_{aq} values at Meerfelder Maar and relatively lower δD_{aq} values at Hässeldala (Supplementary Fig. 4). This pattern is also evident in the $\Delta\delta D_{terr\text{-}aq}$ profiles, which suggests drier conditions at Hässeldala as opposed to more humid conditions at Meerfelder Maar.

The observed opposite patterns of hydroclimate conditions are in agreement with the simulated sea-level pressure field under FIS freshwater forcing presented in Figure 4. As such, Meerfelder Maar and the western sector of Europe lie within a region characterised by relatively lower atmospheric pressure, whereas Hässeldala and Northern Europe are located in an area characterised by relatively higher pressure, thereby explaining the overall differences in summer moisture availability at the two sites. Divergent hydroclimate regimes between Western and Northern Europe are evident in instrumental records (e.g. ref. 36), which highlight a marked North-South contrast of seasonal precipitation during phases of more zonal/meridional atmospheric circulation in the North Atlantic. Altogether, the comparison of the isotope records from Hässeldala and Meerfelder Maar and evidence from model simulations presented in this study support the picture of a spatially complex hydroclimate response to FIS freshwater forcing during the Late Allerød pollen zone/GI1a interval over the North Atlantic.

The first drainage of the Baltic Ice Lake

The deglaciation history of the Fennoscandian Ice Sheet (FIS) in southern Sweden is well known and documented (e.g. ref. 14, 37, 38). During the Late Allerød pollen zone, at ~13000 yr BP, the ice front was located at the southern margin of the south-central Swedish low-land area. Here, owing to the isostatic depression, the land surface was considerably lower than the ice-free lowermost threshold region further south in the Öresund Strait. Since the Baltic Ice Lake (BIL) was dammed up 5-10 m a.s.l. prior to ~13000 yr BP, rapid deglaciation of this southern margin at the water divide near Mt. Billingen during the Late Allerød pollen zone generated a spillway system that connected the BIL to the sea in the west. As such, the rapid retreat of the FIS resulted in a 5-10 m lowering of the BIL also referred to as the first drainage of the BIL[37].

Although this drainage hypothesis has been debated, new reconstructions now suggest that a catastrophic outflow of freshwater actually took place near Mt. Billingen[39]. Unfortunately, these reconstructions lack precise chronological constraints that allow to confidently determining the age of the drainage event. Shore displacement curves for the Mt. Billingen area provide a detailed framework for the timing of the deglaciation near the outlet and the freshwater connection to the sea (e.g. ref. 8, 9). However, a proper effort to systematically calibrate the published radiocarbon dates has never been undertaken, especially since the advent of Bayesian inference to chronological modelling.

Supplementary Methods

Air parcel back trajectories

The trajectory data is an output from the trajectory FLEXPART[40]. FLEXPART is widely used to trace transport of moisture and other chemical species (e.g. ref. 41, 42). Settings and specifications of the simulation used in this study can be found in Viste and Sorteberg[43]. The model was run in a global domain mode filled with 1 million particles and integrated for the time period 1998-2008. Three dimensional wind, temperature and specific humidity from ERA-interim were used to drive the model. To trace the sources of air masses and moisture at Hässeldala, we have defined a target region of 1° by 1° centred over the site (Fig. 1a). We identified the particles arriving to the target region every six hours and traced the particles backward in time for six days to locate their origin. We only traced particles from within the troposphere, assuming that the moisture content of particles above this layer is negligible[44]. The position, mass and specific humidity of these particles were saved during back tracking. To map the starting points of the particles and their path to the target region, the data of the traced particles was re-gridded into 2° by 2° grid boxes. By doing this, the analysis provides the flux of air and water masses that pass through each grid box to reach the target region.

Sediment core alignment

The isotopic data presented in this study refer to Core 5 (ref. 45), whereas the chironomid-based summer temperature reconstruction was obtained on a parallel core, HP4. Core HP4 was obtained in September 2005 close to where previously

studied sediment cores had been sampled[12, 46], using a Russian corer (10 cm diameter, 1 m length). HP4 includes a longer section of the basal unit compared with Core 5 and thus covers a slightly longer time period.

To correlate HP4 to Core 5 we used a Monte Carlo algorithm for core-to-core stratigraphic alignment of proxy records[29] (Supplementary Fig. 5) and then interpolated the reconstructed temperatures to Core 5's sampling resolution using a spline interpolation (the chironomid-based temperature record from HP4 had a relatively higher temporal resolution than the data sets of Core 5). The method involves nonlinear deformation of the entirety of one record onto a reference sequence and makes use of a Monte Carlo technique to deliver the optimal correlation. The approach was designed to correlate proxy records from nearby sedimentary cores and to mutually transfer information between cores retrieved from within the same small depositional basin. We applied our method on loss-on-ignition (LOI) data from both cores. This is an ideal proxy for the application of the matching procedure since LOI time series exhibit the same high-resolution and identifiable stratigraphic pattern of variability in all the cores that have so far been obtained and studied at Hässeldala (e.g ref. 12). This is also due to the relatively small size of the site (approximately 20 m^2).

Using the same procedure, Core 2, which had previously been studied for TOC, pollen and tephra analysis, and which had been dated by 29 ^{14}C dates[46], was correlated to Core 5 (Supplementary Fig. 6). In this case, the cores were correlated using the TOC data after transforming LOI values of Core 5 into TOC as described in

Muschitiello *et al.*[29]. The alignment between these two cores enables transferring a number of the earlier published ^{14}C dates[46] to Core 5 and thus allows us to improve the published chronology of Core 5 (ref. 47), providing the framework for the age-depth model presented in this study and described in the following section.

Bayesian age-depth modelling

By matching the TOC records of Core 2 and Core 5, we were able to transfer additional AMS ^{14}C dates from Core 2 (ref. 46) to the depth scale of Core 5, the template sequence for this study. This procedure resulted in a composite radiocarbon age series of 49 AMS ^{14}C measurements based exclusively on terrestrial plant macro remains (Supplementary Table 1). These 49 ^{14}C dates were then used to establish a Bayesian age-depth relationship, which was modelled using Bacon2.2 (ref. 1) after calibration with the IntCal13 calibration curve[2].

To dismiss spurious dates from the combined ^{14}C age data set, we first modelled the radiocarbon dating sequences of Core 2 and Core 5, separately. The ^{14}C dates for Core 2 and Core 5 were modelled multiple times prescribing different prior information parameters. At this stage we detected four potential age reversals on Core 2 and three on Core 5, which were rejected as probable outlying dates.

We then ran a series of preliminary age models using the composite series of ^{14}C dates in order to identify additional outlying dates. To do so, we approximated the error associated with each radiocarbon measurement to a normal-Gaussian

probability distribution. This approach forces Bacon to become less conciliatory towards outlying ages and helped us to detect two additional outliers.

Finally, a series of 40 out of the 49 initial ^{14}C dates was employed to generate the final model (Supplementary Fig. 7; Supplementary Table 1). We performed several runs until the optimal set up was achieved, i.e. an ideal trade off between coherent prior information parameters and stability of the MCMC simulation. The parameters selected for the model used in this study are the following: thickness *thick*=0.5; accumulation rates *acc.mean*=50 years; gamma distribution of the accumulation rate *acc.shape*=2.5; sample size of the MCMC iterations *ssize*=10,000; related age error t-distribution *t.a*=33, *t.b*=34.

The age-depth model shows a solid structure and appears to be robust during the regional Late Allerød pollen zone and at the transition into the regional Younger Dryas pollen zone (Supplementary Fig. 7). The reliability of the age model is further confirmed by the good agreement between the age of the Allerød-Younger Dryas pollen zone transition (12680 yr BP) at Hässeldala and other regional pollenstratigraphic records in southern Sweden[48].

The age-model results obtained with Bacon were compared to output generated in OxCal4.2 (ref. 4). We run two age models using reasonably ‘relaxed’ *k* parameters of 0.5 and 1, respectively. The parameter defines the stiffness of the model upon the dating sequence when reproducing the sedimentation process. Therefore, the relatively low *k* values adopted constrain the radiocarbon sequence with a sizable degree of flexibility. Furthermore, we have not prescribed any lithological boundary,

which in turn made the model even more flexible. In both cases the age results were equivalent to those yielded by Bacon but with relatively narrower age uncertainties.

Synchronization of NGRIP and IntCal13 time scales

The Greenland Ice Core Chronology 2005 (ref. 49) and the IntCal13 (ref. 2) ^{14}C calibration time scales were matched using the common short-term cosmogenic radionuclide variations in the ice-core ^{10}Be and tree-ring ^{14}C records[50]. The synchronization of the time scales allows overcoming the age uncertainties that accompany the ice-core chronology around the end of Greenland Interstadial 1a and Greenland Stadial 1 (ref. 49) and provides a robust approach for direct chronological comparisons of climate events between ice core and radiocarbon-dated archives.

To produce the synchronized ice-core records displayed in Figures 2 and 3, we applied the transfer function presented in Muscheler *et al.*[50] using the most robust results from regression analyses of windows of 2000-years length, subsequently shifted in steps of 100 years (see Muscheler *et al.*[50] for details). Offset values at 100-year resolution were then linearly interpolated at 20-year resolution in order to assign a specific age offset to each NGRIP $\delta^{18}O$, *d*-excess, and GRIP accumulation data point relative to the IntCal13 time scale. Where offset values were not statistically significant, i.e. around 12000 yr BP, we also used linearly interpolated values. The offset between the time scales should then be regarded as uncertain in this part of the records[50].

Biomarker identification, quantification and hydrogen isotope analysis

The saturated hydrocarbon fraction was analysed by gas chromatography - mass spectrometry (GC-MS) for identification and quantification. GC-MS analysis was performed on a Shimadzu GCMS-QP2010 Ultra equipped with an AOC-20i auto sampler and a split-splitless injector operated in splitless mode. Volatile components were separated using a Zebron ZB-5HT Inferno GC column (30 m length, 0.25 mm inner diameter, 0.25 μm film thickness). The GC oven was programmed to heat with 20 °C min^{-1} starting at 60°C, to 180°C, and subsequently the temperature was ramped to 320 °C at 4 °C min^{-1}, followed by an isothermal phase of 30 min. Helium was used as a carrier gas with a continuous flow rate of 1 ml min^{-1}. The ion source of the MS operating system was set to 200 °C and the ionization energy at 70 eV. C_{18} to C_{33} *n*-alkanes were identified based on mass spectra from the literature and retention times. Concentration of individual compounds was based on the comparison of peak areas relative to that of an internal standard (squalane) that was added to the samples before total lipid extraction.

Hydrogen isotope ratios were determined by gas chromatography-isotope ratio monitoring-mass spectrometry (GC-IRMS) with a Thermo Finnigan Delta XL mass spectrometer coupled to a Trace Ultra GC via a GC Isolink Conflo IV system. A standard mixture of *n*-alkanes with known isotopic composition (reference mixture A4, provided by Arndt Schimmelmann, Indiana University, USA) was run several times daily to calibrate the CO_2 reference gas against which the samples were measured. All analyses were performed in triplicate and results are reported as the mean, and expressed relative to the VSMOW scale.

Concentrations and hydrogen isotope ratios of n-C_{21}, n-C_{27}, n-C_{29}, n-C_{31} alkanes discussed in this study are presented in Supplementary Figure 1 and 2, respectively.

Chironomid-inferred quantitative temperature reconstruction

The core HP4 was sampled every 2 cm for chironomid analyses. Over 100 head capsules were counted for each sample, where possible. In most samples, 1-2 g of sediment was sufficient to obtain over 100 head capsules. Here, volumes processed were 4-6 g, to obtain at least 50 head capsules. Previous studies have demonstrated that 50 head capsules are an adequate minimum to establish species diversity in a sample and to provide reliable temperature estimates[51, 52]. The chironomid larval head capsules were prepared for identification following the procedure in Brooks *et al.*[99]. Head capsules were identified using a compound microscope at x100 to x400 magnification, with reference to Cranston[54], Wiederholm[55], Rieradevall and Brooks[56], and Brooks *et al.*[57].

Mean July air temperatures were derived from the fossil chironomid assemblages using an inference model based on a modern Norwegian calibration dataset consisting of 157 lakes, spanning a mean July air temperature range of 3.5-16 °C, a latitudinal range from 80 °N to 58 °N, an altitudinal range from 0-1600 m, and 142 chironomid taxa (ref. 58 and unpublished data). The calibration data set was standardised to the chironomid taxonomy used in Brooks *et al.*[57]. Mean July air temperature estimates were derived using C2 version 1.4.3 software[59]. The chironomid percentage abundance data was square-root transformed and rare taxa

were down-weighted. The model with the best predictive power was a two-component weighted averaging partial least squares (WA-PLS) inverse regression model, with five outlier lakes removed from the data set to improve the fit. The outlier lakes all contained high percentages of only one or two taxa. Sample-specific errors were estimated by bootstrap cross-validation with 1000 cycles. Root-mean-squared-error of prediction (RMSEP) of the 2-component WA-PLS model was 1.12 °C, the coefficient of determination (r^2) was 0.92 and the maximum bias was 0.77 °C.

Removal of changing ice-volume and isotopic fractionation effects on sedimentary δD_{aq}

To investigate the temporal evolution of 'non-amount' effects[60] on δD of precipitation at Hässeldala (δD_p) - and therefore the input of freshwater to the marine source of precipitation - we removed the effect of changes in global ice volume, condensation temperature and postglacial isostatic uplift on the isotopic composition of δD_{aq}. This was made based on the assumption that our δD_{aq} record captured past variations in the isotopic composition of meteoric water[16, 24, 61] and that if substantial changes owing to evaporative enrichment of lake water δD during the period of focus occurred (i.e. the Late Allerød pollen zone/Greenland Interstadial 1a and the onset of the Younger Dryas pollen zone and Greenland Stadial 1), these operated in the opposite direction of the shifts observed in our δD_{aq} record.

The effect of changing ice volume was scaled from the benthic oxygen isotope stack[62] assuming a global change in $\delta^{18}O$ of 1‰ from the Last Glacial Maximum[63]. The result was then removed from the δD_{aq} record using the equation (1):

(1)

$$\delta D_{aq-ocean} = \left[\frac{1000 + \delta D_{aq}}{(8 \times 0.001 \times \delta^{18}O_{ocean}) + 1}\right] - 1000$$

The effect of elevation changes in Blekinge was inferred from a glacial rebound model[64] assuming a linear uplift of ~34 m during the interval 13500-11700 yr BP with a lapse rate of -0.04‰ m^{-1} (cf. ref. 65).

The effect of changing temperature on summer precipitation δD was established using the modern sensitivity of stable isotope values of local precipitation to temperature. The modern annual δD_p-temperature relationship was inferred using monthly weighted mean δD_p and mean temperature data estimated with the Online Isotopes in Precipitation Calculator (OIPC; ref. 3; reference period: 1960-present) and from ERA-interim reanalysis (reference period: 1979-2013), respectively. The choice of using model-based output was driven by the lack of observational δD_p from nearby meteorological stations. We therefore decided to consistently employ reanalysis and assimilation data to determine the local modern δD_p-temperature relationship. The reliability of the δD_p and temperature output was tested by means of comparison between model-based and observational $\delta^{18}O$ of precipitation ($\delta^{18}O_p$) and temperature from the station of Smedby (~85 km NE of Hässeldala). The

comparison shows a good agreement between the predicted and observed annual $\delta^{18}O_p$-temperature relationship (Supplementary Fig. 8).

We underscore that the predicted precipitation water isotope ratios generated with OIPC are based on a statistical model that uses as independent variables only latitudes and elevation[3]. This means that the temporal isotope pattern produced by the OIPC model does not depend on mean annual temperature, making the model-based $\delta^{18}O_p$-temperature relationship between precipitation isotope ratios and temperatures unbiased.

The shortage of observational δD_p data from the study area and its surroundings prevented us from constraining the summer δ-temperature relation. Since our lipid-based δD and chironomid-based temperature reconstructions reflect summer rather than annual conditions, a modern summer δD_p-temperature relationship would have provided a more direct means for comparison with the reconstructions. However, it is well established that in Europe summer and annual δ_p-temperatures are linearly dependent, with the summer relation being generally weaker owing to moisture transpiration from the soil during warm climatic conditions (e.g. ref. 66). Therefore, deviations of the proxy-based summer δD_p-temperature relationship from the modern annual δD_p-temperature relation should be regarded as conservative estimates of the isotopic shifts associated with 'non-amount' effects and namely as an upper limit to the reconstructed evolution of the annual deviation from the δD_p-temperature relation at Hässeldala.

Support for the validity of our δD record corrected for changing ice volume and fractionation effects (δD_{corr}) is offered by another independent isotopic reconstruction. We observe that the ~20‰ drop in δD_{corr} values during Greenland Interstadial 1a, which corresponds to ~2.5‰ in the $\delta^{18}O$ composition of the marine moisture source, is consistent with the 2.4‰ decline in $\delta^{18}O$ recorded by benthic foraminifera from the southwest coast of Sweden[67].

Supporting chronology for the first drainage of the Baltic Ice Lake

We combined the age probability of a number of published radiocarbon dates indicating lake isolations from the sea near 11,000 ^{14}C years BP. The lake isolations were a result of hydro-isostatic unloading associated with deglaciation north of Mt. Billingen. We also compiled two isolation dates from Blekinge, southern Sweden, and one date from the Arkona Basin, southern Baltic Sea, both indicating a lowering of the BIL associated with the drainage (Supplementary Table 2). The dates were selected for the present analysis according to the assessment provided by the authors in their original publications. Combination of the age probability was achieved using the 'R-combine' function in OxCal4.2 (ref. 4) after calibration with the IntCal13 radiocarbon calibration curve[2] (Supplementary Fig. 9).

A potential pitfall of our approach is that all the ^{14}C dates used here are based on bulk sediment. In fact, the isolation of the lake basins was associated with sparse or no vegetation after ice sheet retreat and thus no plant macrofossils were available for radiocarbon dating[8, 9]. Radiocarbon dating on bulk material may be biased owing

to contamination of bulk sediment by different sources of carbon, which can carry significantly younger/older ^{14}C signatures[68]. Nonetheless, this drawback can be circumvented since a well-established stratigraphic relationship exists between the isolation of lake basins west of Mt. Billingen and specific traits featured in the regional pollen stratigraphies[7]. Therefore, the opening of the outlet area at Mt. Billingen can be indirectly and independently dated in other regional sedimentary records where plant macro remains are available for ^{14}C measurements, thus providing a countercheck on the validity of the age results yielded by bulk dates. Moreover, the available ^{14}C date from the Arkona Basin[10] is based on plant macro remains and it therefore provides a direct test of the reliability of the bulk dates. The isolation of several lake basins west of Mt. Billingen is thought to have occurred during the later stage of the Allerød regional pollen zone, corresponding with a phase of distinct increase in NAP values caused by increasing *Artemisia* pollen percentages[7]. This phase has been radiocarbon dated in other sedimentary sequences using plant macro remains and the inferred age is in excellent agreement with that obtained from bulk material elsewhere (Supplementary Table 2).

Transient climate model simulation

The location and the rate of the meltwater discharge have substantial uncertainties during the last deglaciation. In the simulation of the Transient Climate of the Last 21,000 Years (TraCE-21ka; ref. 69, 70, 71) meltwater forcing was derived from the reconstructions of its impact on climate using reconstructions of *i*) Greenland surface

air temperature, *ii*) the Atlantic Meridional Overturning Circulation (AMOC) and the sea level rise as the main constraints for the meltwater forcing, and *iii*) geological indicators of ice sheet retreat and meltwater discharge. We report in Supplementary Table 3 the meltwater fluxes and locations for the period analysed. For more details the reader can refer to He[70].

In the model analysis we selected – as a template for the slow increasing amount of freshwater from the FIS (~13100-12880 yr BP) seen in our proxy records – two periods of 50 years. These intervals are used as a template for the transition into Greenland Interstadial 1a: before the rapid cooling (13000-12951 model yr BP) and during the cooling (12940-12891 model yr BP) at Hässeldala (Supplementary Fig. 10). The abrupt cooling at the proxy location coincides with the end of the simulated meltwater pulse in the Nordic Seas (55°-75°N – 15°W-30°E) that occurred in the model between 13870 and 12900 model yr BP. The consistency of the results was tested analysing the cooling period at the end of the meltwater pulse in the Nordic Seas with respect to the period in the model without or very little meltwater forcing (14350-14000 model yr BP; Supplementary Fig. 11).

Unfortunately, in the transient simulation the simulated freshwater pulse from the FIS is quite low: only 1 m kyr^{-1}: 1 meter of equivalent global sea level rise per thousand year, i.e. 0.011 Sverdrup (1 Sv = 10^6 m^3 s^{-1}). In the TraCE simulation most of the freshwater pulse of the Late Allerød occurs over the Gulf of Mexico (4 m kyr^{-1}) and in the Ross Sea in the Southern Hemisphere (5 m kyr^{-1}). The model shows the largest sensitivity of the AMOC to a freshwater pulse in the Nordic Seas (whereas the

lowest in the Northern Hemisphere for the Gulf of Mexico). Hence, this experiment can still provide valuable insights on the effects of Fennoscandian-sourced freshwater on changes in atmospheric dynamics that in turn impact the signal recorded in proxy reconstructions. On the other hand, given the large uncertainties on quantifying the location and the rate of the meltwater discharge, our proxy data can enlighten and better account for the importance of freshwater forcing from the FIS during the Late AL and at the onset of the Younger Dryas.

Supplementary References

1. Blaauw, M. & Christeny, J. A. Flexible paleoclimate age-depth models using an autoregressive gamma process. *Bayesian Analysis* **6,** 457–474 (2011).

2. Reimer, P. J. *et al.* IntCal13 and Marine13 radiocarbon age calibration curves 0-50,000 years cal BP. *Radiocarbon* **55,** 1869–1887 (2013).

3. Bowen, G. J. & Revenaugh, J. Interpolating the isotopic composition of modern meteoric precipitation. *Water Resources Research* **39,** (2003).

4. Bronk Ramsey, C. OxCal Program, v. 4.1. 7, Radiocarbon accelerator unit, University of Oxford, UK. (2010).

5. Björck, S. Late Weichselian stratigraphy of Blekinge, SE Sweden, and water level changes in the Baltic Ice Lake. University of Lund, Department of Quaternary Geology. *LUNDQUA Thesis* ***1***, 248 (1979).

6. Björck, S. & Digerfeldt, G. Late Weichselian–Early Holocene shore displacement west of Mt. Billingen, within the Middle Swedish end-moraine zone. Boreas **15**, 1–18 (1986).

7. Björck, S. & Digerfeldt, G. Allerød ‐ Younger Dryas sea level changes in southwestern Sweden and their relation to the Baltic Ice Lake development. *Boreas* **20,** 115–133 (1991).

8. Björck, S. & Digerfeldt, G. New 14C dates from Hunneberg supporting the revised deglaciation chronology of the Middle Swedish end moraine zone. *GFF* **103,** 395–404 (1982).

9. Björck, S. & Digerfeldt, G. Late Weichselian shore displacement at Hunneberg, southern Sweden, indicating complex uplift. *GFF* **104,** 131–155 (1982).

10. Bennike, O. & Jensen, J. B. A Baltic Ice Lake lowstand of latest Allerød age in the Arkona Basin, southern Baltic Sea. *Geol. Surv. Denmark Greenl. Bull* **28,** 17–20 (2013).

11. Rao, Z., Jia, G., Qiang, M. & Zhao, Y. Assessment of the difference between mid- and long chain compound specific δDn-alkanes values in lacustrine sediments as a paleoclimatic indicator. *Organic Geochemistry* **76,** 104–117 (2014).

12. Wohlfarth, B. *et al.* Constraining the age of Lateglacial and early Holocene pollen zones and tephra horizons in southern Sweden with Bayesian probability methods. *Journal of Quaternary Science* **21,** 321–334 (2006).

13. Berglund, B. E. *Late-Quaternary vegetation in eastern Blekinge, south-eastern Sweden: A pollen-analytical study*. (Almqvist & Wiksell, 1966).

14. Björck, S. & Möller, P. Late Weichselian environmental history in southeastern Sweden during the deglaciation of the Scandinavian ice sheet. *Quaternary research* **28,** 1–37 (1987).

15. Ficken, K. J., Li, B., Swain, D. L. & Eglinton, G. An n-alkane proxy for the sedimentary input of submerged/floating freshwater aquatic macrophytes. *Organic Geochemistry* **31,** 745–749 (2000).

16. Aichner, B., Herzschuh, U., Wilkes, H., Vieth, A. & Böhner, J. δD values of n-alkanes in Tibetan lake sediments and aquatic macrophytes - A surface sediment study and application to a 16ka record from Lake Koucha. *Organic Geochemistry* **41,** 779–790 (2010).

17. Gao, L., Hou, J., Toney, J., MacDonald, D. & Huang, Y. Mathematical modeling of the aquatic macrophyte inputs of mid-chain n-alkyl lipids to lake sediments: implications for interpreting compound specific hydrogen isotopic records. *Geochimica et Cosmochimica Acta* **75,** 3781–3791 (2011).

18. Kylander, M. E., Klaminder, J., Wohlfarth, B. & Löwemark, L. Geochemical responses to paleoclimatic changes in southern Sweden since the late glacial:

the Hässeldala Port lake sediment record. *Journal of paleolimnology* **50,** 57–70 (2013).

19. Spencer, D. F., Ryan, F. J. & Ksander, G. G. Construction costs for some aquatic plants. *Aquatic Botany* **56,** 203–214 (1997).

20. Aichner, B., Herzschuh, U. & Wilkes, H. Influence of aquatic macrophytes on the stable carbon isotopic signatures of sedimentary organic matter in lakes on the Tibetan Plateau. *Organic Geochemistry* **41,** 706–718 (2010).

21. Eglinton, G. & Hamilton, R. J. Leaf epicuticular waxes. *Science* **156,** 1322–1335 (1967).

22. Diefendorf, A. F., Freeman, K. H., Wing, S. L. & Graham, H. V. Production of n-alkyl lipids in living plants and implications for the geologic past. *Geochimica et Cosmochimica Acta* **75,** 7472–7485 (2011).

23. Maffei, M. Chemotaxonomic significance of leaf wax alkanes in the gramineae. *Biochemical Systematics and Ecology* **24,** 53–64 (1996).

24. Sachse, D. *et al.* Molecular Paleohydrology: Interpreting the Hydrogen-Isotopic Composition of Lipid Biomarkers from Photosynthesizing Organisms. *Annual Review of Earth and Planetary Sciences* **40,** 221–249 (2012).

25. Schwinning, S., Davis, K., Richardson, L. & Ehleringer, J. Deuterium enriched irrigation indicates different forms of rain use in shrub/grass species of the Colorado Plateau. *Oecologia* **130,** 345–355 (2002).

26. Bush, R. T. & McInerney, F. A. Leaf wax n-alkane distributions in and across modern plants: Implications for paleoecology and chemotaxonomy. *Geochimica et Cosmochimica Acta* **117,** 161–179 (2013).

27. Ampel, L., Kylander, M. E., Steinthorsdottir, M. & Wohlfarth, B. Abrupt climate change and early lake development–the Lateglacial diatom flora at Hässeldala Port, southeastern Sweden. *Boreas* **44,** 94–102 (2015).

28. Sachse, D., Radke, J. & Gleixner, G. δD values of individual n-alkanes from terrestrial plants along a climatic gradient - Implications for the sedimentary biomarker record. *Organic Geochemistry* **37,** 469–483 (2006).

29. Muschitiello, F., Andersson, A., Wohlfarth, B. & Smittenberg, R. H. The C 20 highly branched isoprenoid biomarker–A new diatom-sourced proxy for summer trophic conditions? *Organic Geochemistry* **81,** 27–33 (2015).

30. Uchikawa, J., Popp, B. N., Schoonmaker, J. E. & Xu, L. Direct application of compound-specific radiocarbon analysis of leaf waxes to establish lacustrine sediment chronology. *Journal of Paleolimnology* **39,** 43–60 (2008).

31. Hou, J. *et al.* Radiocarbon dating of individual lignin phenols: A new approach for establishing chronology of late quaternary lake sediments. *Analytical Chemistry* **82,** 7119–7126 (2010).

32. Jones, T. D. *et al.* Diatom-inferred late Pleistocene and Holocene palaeolimnological changes in the Ioannina basin, northwest Greece. *Journal of Paleolimnology* **49,** 185–204 (2013).

33. Douglas, P. M. J. *et al.* Pre-aged plant waxes in tropical lake sediments and their influence on the chronology of molecular paleoclimate proxy records. *Geochimica et Cosmochimica Acta* **141,** 346–364 (2014).

34. Smittenberg, R. H., Eglinton, T. I., Schouten, S. & Damsté, J. S. S. Ongoing buildup of refractory organic carbon in boreal soils during the Holocene. *Science* **314,** 1283–1286 (2006).

35. Rach, O., Brauer, a., Wilkes, H. & Sachse, D. Delayed hydrological response to Greenland cooling at the onset of the Younger Dryas in western Europe. *Nature Geoscience* **7,** 109–112 (2014).

36. Langebroek, P. M., Werner, M. & Lohmann, G. Climate information imprinted in oxygen-isotopic composition of precipitation in Europe. *Earth and Planetary Science Letters* **311,** 144–154 (2011).

37. Björck, S. A review of the history of the Baltic Sea, 13.0-8.0 ka BP. *Quaternary International* **27,** 19–40 (1995).

38. Lundqvist, J. & Wohlfarth, B. Timing and east-west correlation of south Swedish ice marginal lines during the Late Weichselian. *Quaternary Science Reviews* **20,** 1127–1148 (2001).

39. Swärd, H., O´Regan, M., Ampel, L., Ananyev, R., Chernykh, D., Floden, T., Greenwood, S.L., Kylander, M.E., Mörth, C.M., Preto, P. & Jakobsson, M. Regional deglaciation and postglacial lake development as reflected in a 74 m sedimentary record from Lake Vättern, southern Sweden. *GFF*, 1-19 (2015).

40. Stohl, A., Forster, C., Frank, A., Seibert, P. & Wotawa, G. Technical note: The Lagrangian particle dispersion model FLEXPART version 6.2. *Atmospheric Chemistry and Physics Discussions* **5,** 4739–4799 (2005).

41. Nieto, R., Durán-Quesada, A. M. & Gimeno, L. Major sources of moisture for Antarctic ice-core sites identified through a Lagrangian approach. *Clim Res* **40,** 45–49 (2010).

42. Warneke, C. *et al.* An important contribution to springtime Arctic aerosol from biomass burning in Russia. *Geophysical Research Letters* **37,** (2010).

43. Viste, E. & Sorteberg, A. Moisture transport into the Ethiopian highlands. *International journal of climatology* **33,** 249–263 (2013).

44. Urban, J. *et al.* Global observations of middle atmospheric water vapour by the Odin satellite: An overview. *Planetary and Space Science* **55,** 1093–1102 (2007).

45. Steinthorsdottir, M., Wohlfarth, B., Kylander, M. E., Blaauw, M. & Reimer, P. J. Stomatal proxy record of CO2 concentrations from the last termination suggests an important role for CO2 at climate change transitions. *Quaternary Science Reviews* **68,** 43–58 (2013).

46. Davies, S. M. *et al.* Were there two Borrobol Tephras during the early Lateglacial period: Implications for tephrochronology? *Quaternary Science Reviews* **23,** 581–589 (2004).

47. Steinthorsdottir, M. *et al.* Synchronous records of pCO2 and δ14C suggest rapid, ocean-derived pCO2 fluctuations at the onset of Younger Dryas. *Quaternary Science Reviews* **99,** 84–96 (2014).

48. Muschitiello, F. & Wohlfarth, B. Time-transgressive environmental shifts across Northern Europe at the onset of the Younger Dryas. *Quaternary Science Reviews* **109,** 49–56 (2015).

49. Rasmussen, S. O. *et al.* A new Greenland ice core chronology for the last glacial termination. *Journal of Geophysical Research: Atmospheres* **111,** D061202 (2006).

50. Muscheler, R., Adolphi, F. & Knudsen, M. F. Assessing the differences between the IntCal and Greenland ice-core time scales for the last 14,000 years via the common cosmogenic radionuclide variations. *Quaternary Science Reviews* **106,** 81–87 (2014).

51. Heiri, O. & Lotter, A. F. Effect of low count sums on quantitative environmental reconstructions: An example using subfossil chironomids. *Journal of Paleolimnology* **26,** 343–350 (2001).

52. Quinlan, R. & Smol, J. P. Setting minimum head capsule abundance and taxa deletion criteria in chironomid-based inference models. *Journal of Paleolimnology* **26,** 327–342 (2001).

53. Brooks, S. J., Mayle, F. & Lowe, J. Chironomid ‐ based Lateglacial climatic reconstruction for southeast Scotland. *Journal of Quaternary Science* **12,** 161–167 (1997).

54. Cranston, P. S. *A key to the larvae of the British Orthocladiinae (Chironomidae)*. (Freshwater Biological Association Ambleside, 1982).

55. Wiederholm, T. Chironomidae of the Holarctic region. Keys and diagnoses. Part 3. Adult males. *Entomologica Scandinavica, Supplement* (1989).

56. Rieradevall, M. & Brooks, S. J. An identification guide to subfossil Tanypodinae larvae (Insecta: Diptera: Chironomidae) based on cephalic setation. *Journal of Paleolimnology* **25,** 81–99 (2001).

57. Brooks, S. J., Langdon, P. G., Heiri, O. & Association, Q. R. *The identification and use of Palaearctic Chironomidae larvae in palaeoecology*. (Quaternary Research Association, 2007).

58. Brooks, S. J. & Birks, H. J. B. Chironomid-inferred air temperatures from Lateglacial and Holocene sites in north-west Europe: Progress and problems. in *Quaternary Science Reviews* **20,** 1723–1741 (2001).

59. Juggins, S. C2 version 1.4.3. University of Newcastle, UK, (2006).

60. Dansgaard, W. Stable isotopes in precipitation. *Tellus A* (1964).

61. Sachse, D., Radke, J. & Gleixner, G. Hydrogen isotope ratios of recent lacustrine sedimentary n-alkanes record modern climate variability. *Geochimica et Cosmochimica Acta* **68,** 4877–4889 (2004).

62. Lisiecki, L. E. & Raymo, M. E. A Pliocene-Pleistocene stack of 57 globally distributed benthic δ18O records. *Paleoceanography* **20,** 1–17 (2005).

63. Schrag, D. P., Hampt, G. & Murray, D. W. Pore Fluid Constraints on the Temperature and Oxygen Isotopic Composition of the Glacial Ocean. *Science* **272,** 1930–1932 (1996).

64. Lambeck, K. Shoreline displacements in southern-central Sweden and the evolution of the Baltic Sea since the last maximum glaciation. *Journal of the Geological Society* **156,** 465–486 (1999).

65. Hammarlund, D., Barnekow, L., Birks, H. J. B., Buchardt, B. & Edwards, T. W. D. Holocene changes in atmospheric circulation recorded in the oxygen-isotope stratigraphy of lacustrine carbonates from northern Sweden. *The Holocene* **12,** 339–351 (2002).

66. Field, R. D. Observed and modeled controls on precipitation δ18O over Europe: From local temperature to the Northern Annular Mode. *Journal of Geophysical Research: Atmospheres* **115,** (2010).

67. Bodén, P., Fairbanks, R. G., Wright, J. D. & Burckle, L. H. High ‐ resolution stable isotope records from southwest Sweden: The drainage of the Baltic Ice Lake and Younger Dryas Ice Margin Oscillations. *Paleoceanography* **12,** 39–49 (1997).

68. Björck, S. & Wohlfarth, B. in *Tracking environmental change using lake sediments* 205–245 (Springer, 2001).

69. Liu, Z. *et al.* Transient simulation of last deglaciation with a new mechanism for Bolling-Allerod warming. *Science* **325,** 310–314 (2009).

70. He, F. Simulating transient climate evolution of the last deglaciation with CCSM3. (2011).

71. He, F. *et al.* Northern Hemisphere forcing of Southern Hemisphere climate during the last deglaciation. *Nature* **494,** 81–5 (2013).
